# The Tumor Suppressor TPD52‐Governed Endoplasmic Reticulum Stress is Modulated by APC^Cdc20^

**DOI:** 10.1002/advs.202405441

**Published:** 2024-10-14

**Authors:** Weichao Dan, Yizeng Fan, Yuzhao Wang, Tao Hou, Yi Wei, Bo Liu, Mengxing Li, Jiaqi Chen, Qixiang Fang, Taotao Que, Yuzeshi Lei, Chendong Guo, Chi Wang, Yang Gao, Jin Zeng, Lei Li

**Affiliations:** ^1^ Department of Urology The First Affiliated Hospital of Xi'an Jiaotong University Xi'an 710061 P. R. China; ^2^ Key Laboratory for Tumor Precision Medicine of Shaanxi Province The First Affiliated Hospital of Xi'an Jiaotong University Xi'an 710061 P. R. China; ^3^ Key Laboratory of Environment and Genes Related to Diseases Ministry of Education Xi'an 710061 P. R. China

**Keywords:** ATF6, Cdc20, ER stress, TPD52, unfolded protein response

## Abstract

Aberrant regulation of unfolded protein response (UPR)/endoplasmic reticulum (ER) stress pathway is associated with cancer development, metastasis, and relapse, and the UPR signal transducer ATF6 has been proposed as a diagnostic and prognostic marker for many cancers. However, a causal molecular link between ATF6 activation and carcinogenesis is not established. Here, it is found that tumor protein D52 (TPD52) integrates ER stress and UPR signaling with the chaperone machinery by promoting S2P‐mediated cleavage of ATF6. Although TPD52 has been generally considered as an oncogene, TPD52 is identified as a novel tumor suppressor in bladder cancer. Significantly, attenuation of the ER stress via depletion of TPD52 facilitated tumorigenesis in a subset of human carcinomas. Furthermore, the APC^Cdc20^ E3 ligase is validated as the upstream regulator marking TPD52 for polyubiquitination‐mediated proteolysis. In addition, inactivation of Cdc20 sensitized cancer cells to treatment with the ER stress inducer in a TPD52‐dependent manner. Thus, the study suggests that TPD52 is a novel Cdc20 substrate that may modulate ER stress to prevent tumorigenesis.

## Graphical abstract

This study found that tumor protein D52 (TPD52) integrates ER stress by promoting S2P‐mediated cleavage of ATF6, thus inhibiting tumor progression. Furthermore, the APC^Cdc20^ E3 ligase is identified as the upstream regulator marking TPD52 for polyubiquitination‐mediated proteolysis. Inactivation of Cdc20 sensitized cancer cells to treatment with the ER stress inducer in a TPD52‐dependent manner.

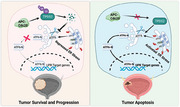

## 1 Introduction

The endoplasmic reticulum (ER) contributes to the initial protein maturation and proper folding of proteins in most eukaryotic cells. Upon either physiological or pathological alterations in ER homeostasis, unfolded or misfolded proteins accumulate in the ER lumen, a condition also referred to as ER stress.^[^ ^1^ ^]^ In response to ER stress, a series of complementary adaptations to address protein‐folding alterations, referred to as the unfolded protein response (UPR), is activated in cells.^[^ ^2^ ^]^ However, when cells are irreversibly exposed to ER stress above a certain threshold, the UPR triggers apoptosis to eliminate damaged cells. The UPR signaling pathway elicits multiple cellular responses mediated by the activation of at least three major ER stress sensors located on the ER membrane: Inositol‐requiring enzyme type 1 (IRE1) (both α and β isoforms), activating transcription factor 6 (ATF6) (both α and β isoforms) and protein kinase RNA‐like ER kinase (PERK).^[^ ^3^, ^4^ ^]^ In the absence of ER stress, Bip (also called GRP78 and HSPA5) preferentially participates in inhibitory interactions with ER stress sensors. Under ER stress, ATF6 traffics from the ER to the Golgi apparatus, where it is cleaved sequentially by site‐1 protease (S1P) and site‐2 protease (S2P) to release a soluble basic‐leucine zipper (bZIP) transcription factor, ATF6‐N terminal.^[^ ^5^ ^]^ Studies have confirmed that Bip release allows ATF6 to be transported to the Golgi via the formation of coat protein II (COPII)‐coated vesicles.^[^ ^6^ ^]^ Moreover, hypoglycosylated ATF6 fails to interact with Calreticulin, resulting in more rapid transport.^[^ ^7^ ^]^ ATF6 also exists as a mixture of disulfide‐linked monomers and oligomers, which can be regulated by ERp18.^[^ ^8^, ^9^ ^]^ However, the regulatory mechanisms underlying the stress‐specific activation of ATF6 during the determination of cell fate remain unclear.

TPD52 isoform 3, a member of the TPD52 (tumor protein D52) family, appears to be involved in trafficking via the exo‐ and endocytic pathways.^[^ ^10^ ^]^ In addition, evidence has shown that phosphorylation‐dependent regulation of TPD52 in turn regulates endolysosomal trafficking in secretory cell types.^[^ ^11^ ^]^ In cancers, accumulating evidence indicates an oncogenic role for TPD52. Consistent with this idea, multiple studies have confirmed the overexpression of TPD52 in several cancers.^[^ ^12^, ^13^, ^14^, ^15^, ^16^ ^]^ Recently, we convincingly showed that TPD52 isoform 1 acts as a potential proto‐oncogene by activating the chaperone‐mediated autophagy pathway.^[^ ^17^ ^]^ In contrast, TPD52 was identified as a potential tumor suppressor in hepatocellular carcinoma.^[^ ^18^ ^]^ These inconsistent findings indicate a cell‐ or tissue‐context‐dependent function of TPD52 in the malignant progression of cancer.

Here, we demonstrated that TPD52 acts as a tumor suppressor in bladder cancer. Under unresolved ER stress, TPD52 activation leads to excessive accumulation of intracellular ER stress by facilitating ATF6 cleavage in the Golgi apparatus, resulting in excessive apoptosis. In parallel, APC^Cdc20^ promotes TPD52 polyubiquitination at K179 and its subsequent degradation. Thus, our findings reveal de novo TPD52/ATF6‐mediated signaling activated during ER stress, which is abrogated by APC^Cdc20^‐mediated polyubiquitination of TPD52 at K179, and thereby suggests synergistic strategies for cancer therapy.

## 2 Results

### 2.1 TPD52 Promotes ATF6 Activation During the ER Stress

**Figure 1:**
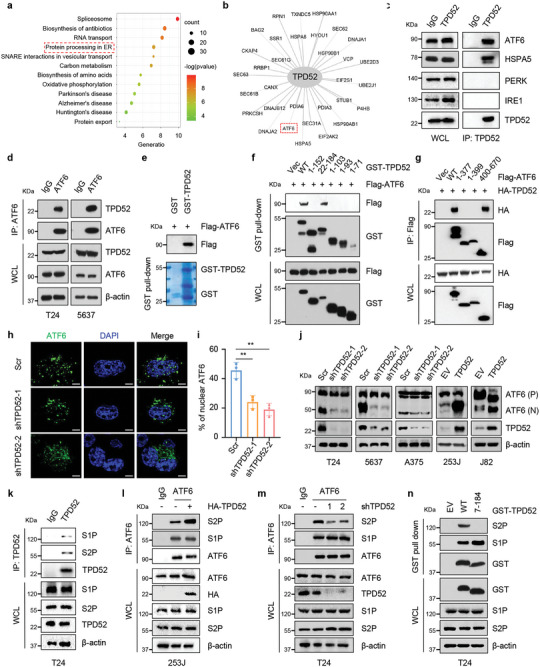
TPD52 promotes ATF6 activation during the UPR. a) Anti‐TPD52 immunoprecipitates (IPs) coupled with mass spectrometry analysis (MS) to identify TPD52‐interacting proteins in T24 cells. IPs‐MS results were subjected to enrichment analysis. b) The proteins were enriched in the “protein processing in endoplasmic reticulum” categories. c) Immunoblot (IB) analysis of whole cell lysates (WCL) and anti‐TPD52 immunoprecipitates (IPs) derived from T24 cells. d) IB analysis of WCL and anti‐ATF6 IPs derived from T24 and 5637 cells. e) GST pull‐down assay revealed the direct interaction between TPD52 and ATF6. The upper panel presents the result of IB by using the antibody against Flag, and the lower Coomassie blue staining showing the gels for purified proteins. f) IB analysis of WCL and GST‐pull‐down products derived from 293T cells transfected with Flag‐ATF6 and indicated constructs of GST‐TPD52. Vec, vector. WT, wild type. g) IB analysis of WCL and anti‐Flag IPs derived from 293T cells transfected with HA‐TPD52 and indicated constructs of Flag‐ATF6. Vec, vector. WT, wild type. h‐i) Representative fluorescence photomicrographs h) and quantification i) of ATF6 in T24 cells obtained via confocal microscopy. Where indicated, 1 µg mL^−1^ tunicamycin (Tu) was added for 6 h before harvesting the cells. Scale bars represent 5 µm. j) IB analysis of WCL derived from T24, 5637, A375, 253J, and J82cells stably expressing indicating plasmids. Where indicated, 1 µg mL^−1^ Tu was added before harvesting the cells. Scr, Scramble. EV, empty vector. ATF6 (P) and (N), respectively, refer to ATF6 precursor and cleavaged form. k) IB analysis of WCL and anti‐TPD52 IPs derived from T24 cells. l) IB analysis of WCL and anti‐ATF6 IPs derived from 253J cells overexpressing TPD52 or EV. Where indicated, 1 µg mL^−1^ Tu was added before harvesting the cells. EV, empty vector. m) IB analysis of WCL and anti‐ATF6 IPs derived from T24 cells stably expressing shTPD52 or Scr. Where indicated, 1 µg ml^−1^ Tu was added before harvesting the cells. Scr, Scramble. n) IB analysis of WCL and GST‐pull‐down products derived from T24 cells transfected with indicated constructs of GST‐TPD52. Where indicated, 1 µg mL^−1^ Tu was added before harvesting the cells. EV, empty vector. WT, wild type.

To further elucidate the molecular functions and underlying mechanisms of TPD52 in cancer, we immunoprecipitated exogenous TPD52 from T24 human bladder cancer cells and identified potential interacting proteins by mass spectrometry (MS) (Figure S1a, Supporting Information). We identified approximately 439 proteins as TPD52‐binding molecules in T24 cells, and Gene Ontology (GO) enrichment analysis revealed that these molecules were involved in protein processing in the endoplasmic reticulum (**Figure** **1**a), suggesting that TPD52 might participate in regulating endoplasmic reticulum (ER) homeostasis. Interestingly, we identified two key ER stress response components, HSPA5 and ATF6, in our MS analysis (Figure 1b). Consistent with the MS results, endogenous TPD52 bound ATF6 and HSPA5 in T24 and 5637 cells, as determined by in vivo reciprocal immunoprecipitation assays (Figure 1c,d). In addition, exogenously expressed HA‐TPD52 and Flag‐ATF6 in 293T cells were reciprocally coimmunoprecipitated (Figure S1b,c, Supporting Information). Furthermore, we observed a direct interaction between TPD52 and ATF6, as determined by a GST affinity isolation assay (Figure 1e). We then generated a series of truncation mutants of TPD52 and ATF6 to identify the precise binding region(s) that facilitate their interaction. The results revealed that the carboxy‐terminus of TPD52 (152‐184) bound to ATF6 (Figure 1f; Figure S1d, Supporting Information). Conversely, the luminal domain of ATF6 (amino acids 400–670) strongly associated with TPD52, whereas the cytosolic domain (amino acids 1–377) and transmembrane domain (amino acids 378–399) of ATF6 completely failed to bind TPD52, suggesting that the region comprising amino acids 400–670 is required for the association of ATF6 with TPD52 (Figure 1g; Figure S1e, Supporting Information).

ATF6 is normally localized in the endoplasmic reticulum (ER) and traffics to the Golgi apparatus after the onset of ER stress for conversion into its active form, which enters the nucleus to induce the expression of target genes.^[^ ^18^ ^, 19]^ As shown in Figure 1h,i and Figure S1f, Supporting Information, TPD52 downregulation decreased ATF6 localization in nucleus after treatment with tunicamycin (Tu; Figure 1h), a pharmacological ER stress inducer that blocks the N‐glycosylation of proteins.^[^ ^20^ ^]^ In addition, we found that TPD52 knockdown markedly reduced the level of cleaved ATF6 (ATF6(N); Figure 1j), whereas overexpression of TPD52 increased the ATF6(N) protein level (Figure 1j), indicating that TPD52 can positively regulate ATF6 processing for its activation during ER stress. Activation of ATF6 requires sequential cleavage by site‐1 protease (S1P) and site‐2 protease (S2P) to release the active transcription factor ATF6(N).^[^ ^21^, ^22^ ^]^ However, neither S1P nor S2P exhibited a change in expression after modulation of TPD52 expression (Figure S1g–i, Supporting Information). Recent studies have shown that TPD52 colocalizes with the Golgi apparatus, the intracellular site where ATF6 is cleaved by S1P/S2P, but not the ER.^[^ ^23^ ^]^ A coimmunoprecipitation assay showed the interactions of TPD52 with S1P/S2P in T24 cells (Figure 1k), leading us to explore the effects of TPD52 on the ER stress‐induced interactions between ATF6 and S1P/S2P. We discovered that the Tu‐induced interaction between ATF6 and S2P but not between ATF6 and S1P was increased by TPD52 overexpression (Figure 1l), whereas it was decreased in TPD52‐knockdown cells (Figure 1m). Thus, our present findings demonstrate that TPD52 promotes ATF6 cleavage during ER stress by increasing the interaction between ATF6 and S2P.

TPD52 (isoform 3) belongs to the tumor protein D52 (TPD52) family and contains an N‐terminal domain different from that of Prostate Leucine Zipper (PrLZ, TPD52 isoform 1), and we further found that PrLZ and ATF6 also interact with each other (Figure S1j,k, Supporting Information). We further found that PrLZ failed to promote ATF6 processing for its activation during ER stress (Figure S1l, Supporting Information). In addition, we confirmed by a coimmunoprecipitation assay that PrLZ can bind only to S1P (Figure S1m, Supporting Information). Interestingly, deletion of TPD52 N‐terminal domain did not disrupt the binding of ATF6 to S1P but disrupted its interaction with S2P (Figure 1n). Overexpression of PrLZ did not increase the interaction between ATF6 and either S1P or S2P (Figure S1n, Supporting Information). Taken together, these results suggest that PrLZ and TPD52 might exert different functions by binding to ATF6 during ER stress (Figure S1o, Supporting Information).

### 2.2 TPD52 Regulates ER Stress and ER Size through ATF6

**Figure 2:**
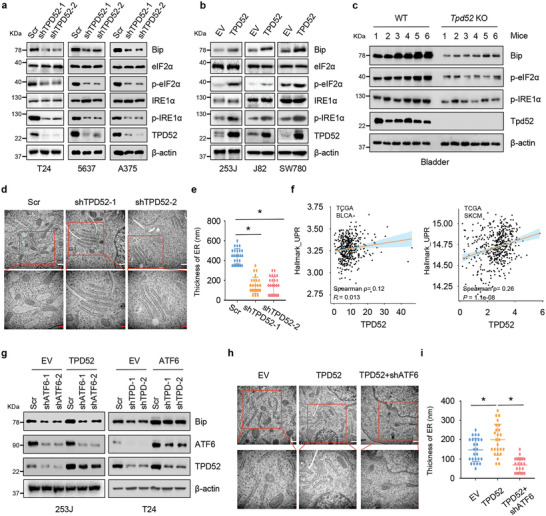
TPD52 regulates UPR signals and ER size through ATF6. a) Immunoblot (IB) analysis of whole cell lysates (WCL) derived from T24, 5637, and A375 cells stably expressing shTPD52 or Scr. Scr, Scramble. b) IB analysis of WCL derived from 253J, J82, and SW780 cells stably overexpressing TPD52 or EV. EV, empty vector. c) IB analysis of the WCL derived from wild‐type (WT) or *Tpd52*‐knockout (*TPD52* ^−/−^) mice bladder tissues. d) Representative transmission electron micrographs (TEMs) image of T24 cells stably expressing shTPD52 or Scr. Where indicated, 1 µg mL^−1^ Tu was added before harvesting the cells. Scr, Scramble. Scale bars: 500 nm (white); 200 nm (red). e) ER thickness was quantified for the indicated TEM samples in (d). f) Spearman analysis of gene‐expression data from patients with bladder cancer (BLCA, TCGA dataset, n = 411 samples) and skin cutaneous melanoma (SKCM, TCGA dataset, n = 472 samples) was used for depicting the correlation between TPD52 and unfolded protein response (UPR). g) IB analysis of WCL derived from 253J ATF6 knockdown or control cells transduced with TPD52 lentivirus or T24 TPD52 knockdown or control cells transduced with ATF6 lentivirus. TPD, TPD52. Scr, Scramble. EV, empty vector. h) Representative TEMs image of T24 ATF6 knockdown or control cells transduced with TPD52 lentivirus. Where indicated, 1 µg mL^−1^ Tu was added before harvesting the cells. Scr, Scramble. EV, empty vector. Scale bars: 500 nm (white); 200 nm (red). **i**. ER thickness was quantified for the indicated TEM samples in (h).

We then aimed to explore the regulatory role of TPD52 in ER stress. As shown in **Figure** **2**a, depletion of endogenous TPD52 by short hairpin RNA (shRNA) decreased the protein levels of the ER chaperone Bip as well as components of the ATF6 branch of the ER stress, including phosphoeIF2α and phosphoIRE1α. In addition, the mRNA levels of Bip and ATF6 were decreased in TPD52‐knockdown T24 and 5637 cells (Figure S2a,b, Supporting Information). The opposite changes were found in cells overexpressing TPD52 (Figure 2b; Figure S2c, Supporting Information). To further validate the roles of TPD52 in ER stress in vivo, we generated *Tpd52* knockout (KO) mice (termed *Tpd52* ^−/‐^ mice) as described before^[^ ^17^ ^]^ (Figure S3a,b, Supporting Information). Notably, significant decreases in the protein levels of Bip, phosphoeIF2α, and phosphoIRE1α in bladders from *Tpd52* ^−/‐^ mice were observed (Figure 2c). In addition, transmission electron microscopy (TEM) showed a decrease in the size of ER compartments in T24 TPD52‐knockdown cells during the Tu‐induced ER stress response (Figure 2d,e). Consistent with our observations, expression of TPD52 was positively correlated with activation of the unfolded protein response (UPR) in the TCGA cohorts of patients with bladder cancer (BCa) or skin cutaneous melanoma (SKCM; Figure 2f). Interestingly, further depletion of endogenous ATF6 in TPD52‐overexpressing T24 cells still resulted in reduced Bip and phosphoIRE1α levels, whereas T24 TPD52 knockdown in cells transfected with ATF6 largely attenuated the downregulation of Bip and phosphoIRE1α (Figure 2g; Figure S2d, Supporting Information). Consistent with this finding, TEM confirmed that ATF6 knockdown decreased the basal ER size in T24 TPD52‐overexpressing cells following Tu treatment (Figure 2h,i), indicating that TPD52 might function mainly through activation of ATF6 and subsequent promotion of ER stress.

We hypothesized the existence of a feedback loop regulating TPD52 protein synthesis during ER stress. Interestingly, we failed to find changes in the protein and mRNA levels of TPD52 in cells with ER stress activation induced by tunicamycin (Tu; Figure S2e–g, Supporting Information). Similarly, TPD52 expression remained stable under treatment with tauroursodeoxycholic acid (TUDCA), which is a well‐known ER stress inhibitor^[^ ^24^, ^25^, ^26^ ^]^ (Figure S2h,i, Supporting Information).

### 2.3 TPD52‐Mediated ER Stress Inhibits Tumorigenesis through ATF6

**Figure 3:**
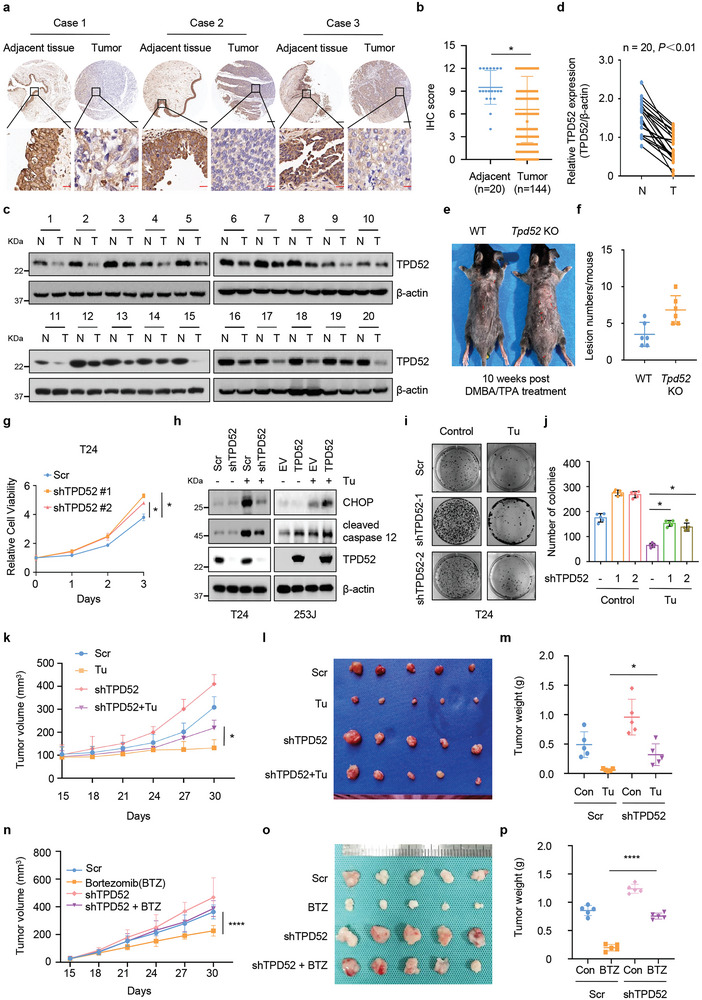
TPD52‐mediated ER stress inhibits tumorigenesis through ATF6. a‐b) Representative IHC images (a) and statistical quantification (b) of TPD52 expression in bladder cancer and normal tissues using immunohistochemical staining. Scale bars: 200 µm (black); 50 µm (red). c) Immunoblot (IB) analysis of whole cell lysates (WCL) of TPD52 protein expression in bladder tumor (T) and paired adjacent normal (N) tissues from 20 bladder cancer patients. d) Quantification of TPD52 protein expression of the blot shown in (c) by using ImageJ software. e‐f) The representative side view (e) and statistical quantification (f) of wild type (WT) or *Tpd52* knockout (*Tpd52* ^−/−^) mice treated with chemical carcinogen (DMBA following with TPA) for 10 weeks (n = 6 for WT mice; n = 6 for *Tpd52* ^−/−^ mice). The neoplasm lesions were arrowed. g) The growth curve of T24 TPD52 knockdown or control cells. Scr, Scramble. *P < 0.05. h) IB analysis of WCL derived from T24 cells stably expressing shTPD52 or Scr and 253J cells stably overexpressing TPD52 or EV. Where indicated, 1 µg mL^−1^ Tu was added before harvesting the cells. Scr, Scramble. EV, empty vector. i‐j) Colony formation assays (i) and quantification (j) of T24 TPD52 knockdown or control cells treated with Tu. Error bars are mean ± s.e.m. *P < 0.05. k‐m) T24 TPD52 knockdown or control cells were subcutaneously injected into nude mice to establish xenograft model and treated with Tu (10 mg kg^−1^, once daily). Statistical analysis of the tumor volumes which were measured every three days and plotted individually (k). Subcutaneous xenograft tumors formed from different groups were dissected (l). Statistical analysis of the weight of the dissected xenografts tumors m). n = 5 mice per experimental group, the results indicate the mean ± S.D. Scr, Scramble. ^*^ *P*<0.05. n‐p) T24 TPD52 knockdown or control cells were subcutaneously injected into nude mice to establish xenograft model and treated with Bortezomib (1 mg kg^−1^, twice a week). Statistical analysis of the tumor volumes which were measured every three days and plotted individually n). Subcutaneous xenograft tumors formed from different groups were dissected o). Statistical analysis of the weight of the dissected xenografts tumors p). n = 5 mice per experimental group, the results indicate the mean ± S.D. Scr, Scramble.

As the expression and role of TPD52 in bladder cancer have not been previously reported, we first evaluated the expression of TPD52 in bladder cancer tissue microarrays. Compared with that in adjacent tissues, TPD52 expression was downregulated in bladder cancer tissues (**Figure** **3**a,b). In addition, lower TPD52 protein levels were observed in 20 bladder cancer tumors (T) compared with the paired adjacent noncancerous tissues (N) (Figure 3c,d). Surprisingly, no impact on body size/weight (Figure S3c,d, Supporting Information) or organ size/weight (Figure S3e,f, Supporting Information) was observed in *Tpd52* ^−/−^ mice compared with wild‐type (WT) mice. To determine whether *Tpd52* knockout accelerates tumorigenesis in vivo, we established a two‐step chemical carcinogen (7,12‐dimethylbenz[a]anthracene (DMBA) followed by 12‐O‐tetradecanoyl phorbol‐13‐acetate (TPA))‐induced skin tumor model as described previously.^[^ ^27^ ^]^ Compared with WT mice, *Tpd52* ^−/−^ mice displayed a higher incidence of skin tumors and increased papilloma burden (Figure 3e,f). Moreover, prognostic analysis of *TPD52* gene using The Cancer Genome Atlas (TCGA) databases confirmed that high levels of *TPD52* were positively correlated with clinical outcomes (Figure S4a, Supporting Information). More importantly, knocking down TPD52 resulted in increases in both cell proliferation (Figure 3g; Figure S4b–e, Supporting Information) and migration (Figure S4f,g, Supporting Information). In contrast, overexpressing TPD52 led to inhibition of both cell proliferation (Figure S4h–j, Supporting Information). Moreover, exogenous ATF6 expression abrogated the tumor‐promoting effects of TPD52 depletion, whereas ATF6 knockdown in cells transfected with TPD52 largely regained its proliferative capacity, suggesting that TPD52 might function through activation of ATF6 and subsequent promotion of ER stress (Figure S4k,l, Supporting Information).

During persistent ER stress, the adaptive UPR might fail to eliminate all unfolded/misfolded proteins, and apoptosis is activated to eliminate the stressed cells.^[^ ^3^, ^4^ ^]^ TPD52 depletion in T24 cells attenuated Tu‐induced ER stress‐associated apoptotic pathway activity compared with that in the control group (Figure 3h; Figure S4m, Supporting Information). Consequently, T24 TPD52‐knockdown cells undergoing ER stress that was induced by Tu treatment grew more robustly (Figure 3i,j) than control cells. In contrast, we found that TPD52 overexpression in 253J cells increased ER stress‐induced apoptosis and proliferative inhibition (Figure 3h; Figure S4n,o, Supporting Information). In keeping with these findings, we discovered that TPD52 knockdown increased the volumes and weights of xenografts under Tu treatment (Figure 3k–m). Bortezomib (BTZ), an antitumor drug that has been used in clinical application, was thought to induce ER stress by inhibiting proteasomal degradation. The xenograft tumor experiments confirmed that depletion of TPD52 conferred resistance to Bortezomib treatment in bladder cancer (Figure 3n–p). Collectively, these results support the idea that TPD52 knockdown contributes to resistance to ER stress‐mediated inhibition of tumor progression both in vitro and in vivo.

### 2.4 The TPD52 Protein Abundance Fluctuates during Cell Cycle Progression and is Negatively Correlated with APC^Cdc20^ Activity

**Figure 4:**
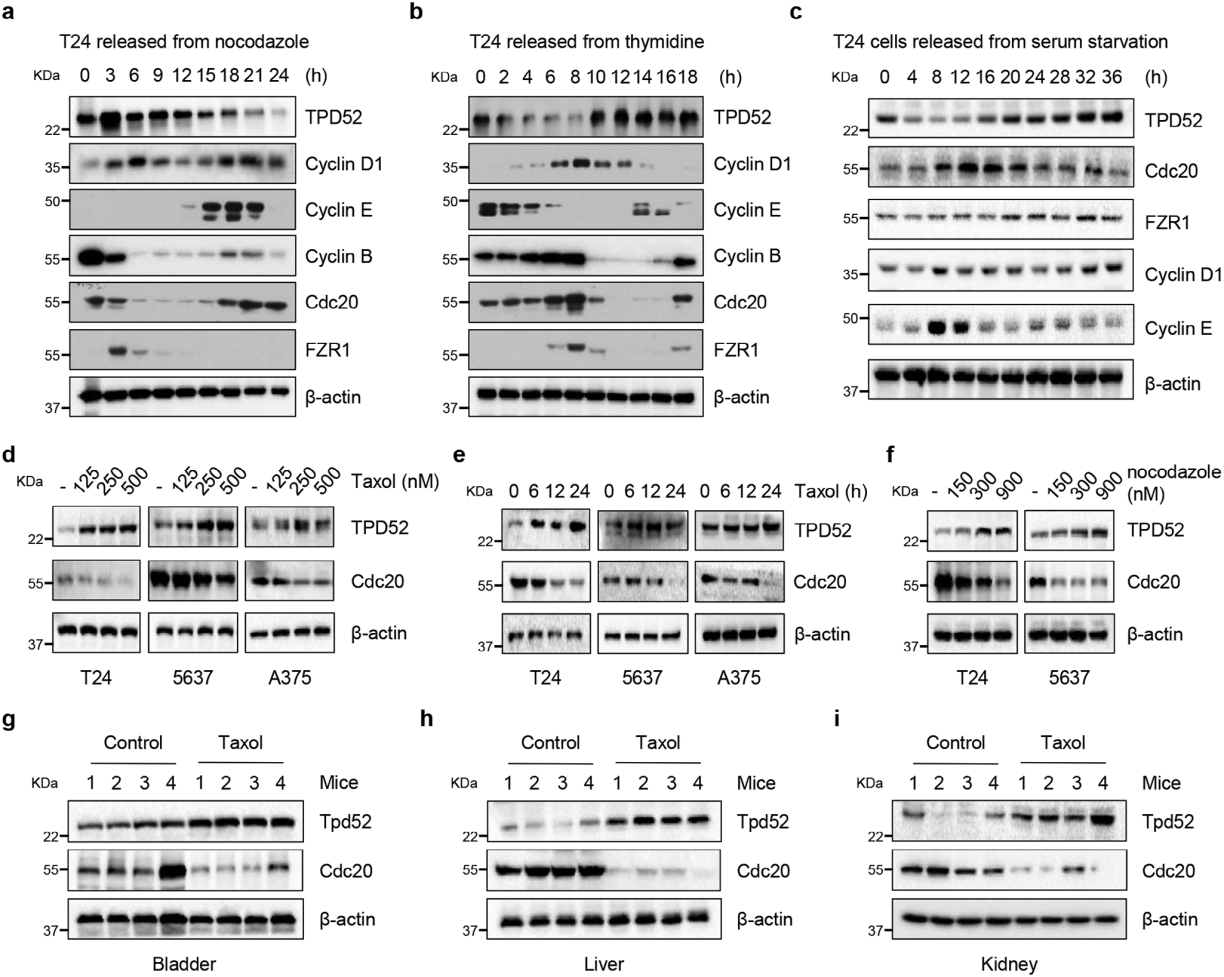
TPD52 protein fluctuates during cell cycle progression. a) Immunoblot (IB) analysis of whole cell lysates (WCL) derived from T24 cells synchronized at M phase by nocodazole block, following by releasing back into the cell cycle for the indicated times. b) IB analysis of WCL derived from T24 cells synchronized at the late G1/S boundary by double‐thymidine block, following by releasing back into the cell cycle for the indicated times. c) IB analysis of WCL derived from T24 cells synchronized at G0 phase by serum starvation block, following by releasing back into the cell cycle for the indicated times. d) IB analysis of WCL derived from T24, 5637, and A375 cells treated with different concentration of Taxol (125, 250 and 500 nM) for 24 h. e) IB analysis of WCL derived from T24, 5637 and A375 cells treated with Taxol (500 nM) for different time (6, 12, and 24 h). f) IB analysis of WCL derived from T24 and 5637 cells treated with different concentration of nocodazole (150, 300 and 900 nM) for 24 h. g‐i) IB analysis of WCL derived from mice bladder (g), liver (h) and kidney (i) tissues treated with Taxol (10 mg kg^−1^) for 7 days.

To explore TPD52‐associated biological processes, we applied gene set enrichment analysis (GSEA) in the TCGA bladder cancer cohort. The results revealed that TPD52 was related to the cell cycle pathway (Figure S5a, Supporting Information). Notably, in our study, the TPD52 protein abundance fluctuated during the cell cycle in cells synchronized by nocodazole treatment, displaying a dramatic reduction when APC^Cdc20^ was most active (**Figure** **4**a; Figure S5b, Supporting Information). Consistent with this finding, an inverse correlation between APC^Cdc20^ activity and the abundance of TPD52 was also observed in cells synchronized by double thymidine block (Figure 4b; Figure S5b, Supporting Information) and serum starvation block (Figure 4c; Figure S5b, Supporting Information).

Previous results demonstrated that paclitaxel (Taxol) and nocodazole can induce G2/M arrest and activate spindle‐assembly‐checkpoint (SAC) to suppress APC^Cdc20^.^[^ ^28^, ^29^ ^]^ A similar reduction in the Cdc20 protein abundance was observed after prolonged mitotic arrest (Figure 4d–f), possibly due partially to increased Cdc20 self‐ubiquitination. Consequently, an elevated TPD52 protein abundance was detected in multiple cancer cell lines (Figure 4d–f). We also observed that Taxol treatment increased the TPD52 protein abundance in various organs of normal mice (Figure 4g–i; Figure S5c, Supporting Information). In summary, all these results indicate that the TPD52 protein abundance fluctuates during cell cycle progression.

### 2.5 Cdc20 Interacts with TPD52 and Promotes its Polyubiquitination and Degradation

**Figure 5:**
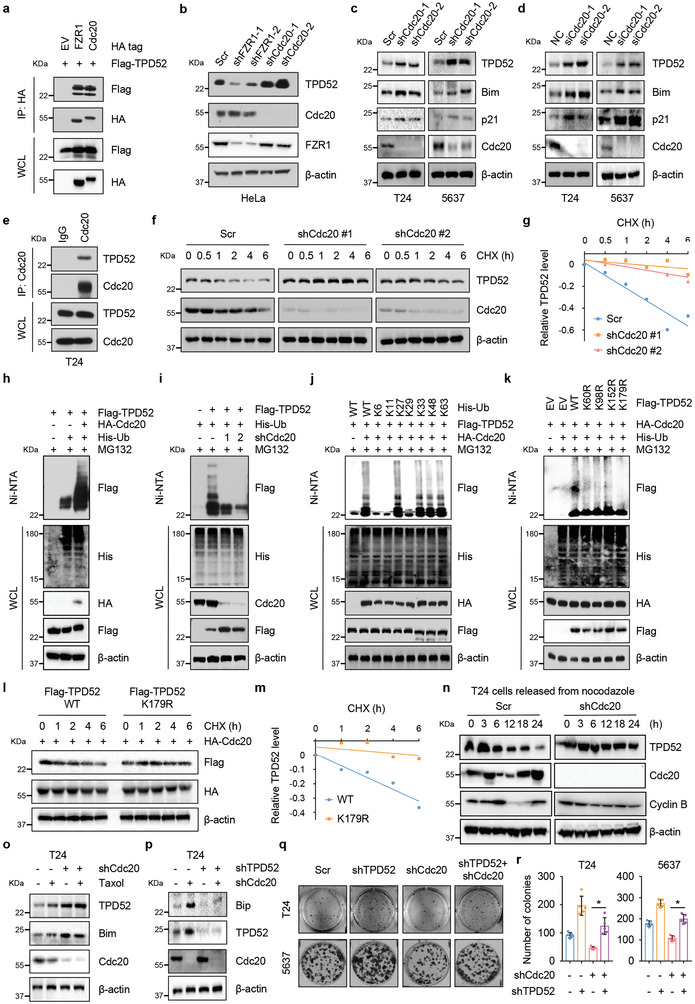
Cdc20 interacts with and promotes TPD52 K48‐linked polyubiquitination and degradation. a) Immunoblot (IB) analysis of whole cell lysates (WCL) and anti‐HA immunoprecipitates (IPs) derived from 293T cells transfected with HA‐FZR1, HA‐Cdc20 and Flag‐TPD52. 30 hours post‐transfection, cells were treated with 20 µM MG132 for 6 hours before harvesting. EV, empty vector. b) IB analysis of WCL derived from HeLa cells stably expressing shFZR1 or shCdc20. Scr, Scramble. c) IB analysis of WCL derived from T24 and 5637 cells stably expressing shCdc20 or Scr. Scr, Scramble. d) IB analysis of WCL derived from T24 and 5637 cells transfected with siCdc20 or NC. NC, negative control. e) IB analysis of WCL and anti‐Cdc20 IPs derived from T24 cells. Cells were treated with 20 µM MG132 for 6 h before harvesting. f) Cdc20 knockdown cells (shCdc20) as well as parental T24 cells (Scr) were treated with 100 µg mL^−1^ cycloheximide (CHX) for the indicated time period before harvesting. Equal amounts of WCL were immunoblotted with the indicated antibodies. Scr, Scramble. g) The TPD52 protein abundance in (f) was quantified by ImageJ and plotted as indicated. TPD52 bands were normalized to β‐actin. h) IB analysis of WCL and Ni‐NTA pull‐down products derived from 293T cells transfected with Flag‐TPD52, HA‐Cdc20 and His‐Ub. Where indicated, 20 µM MG132 was added for 6 h before harvesting the cells. i) IB analysis of WCL and Ni‐NTA pull‐down products derived from T24 cells stably expressing shCdc20 or Scr transfected with Flag‐TPD52 and His‐Ub. Where indicated, 20 µM MG132 was added for 6 h before harvesting the cells. j) IB analysis of WCL and Ni‐NTA pull‐down products derived from 293T cells transfected with Flag‐TPD52, HA‐Cdc20 and the indicated K‐only ubiquitin mutants. Where indicated, 20 µM MG132 was added for 6 h before harvesting the cells. k) IB analysis of WCL and Ni‐NTA pull‐down products derived from 293T cells transfected with Flag‐tagged wild type (WT) and mutated TPD52 (K60R, K98R, K152R, and K179R), HA‐Cdc20 and His‐Ub. Where indicated, 20 µM MG132 was added for 6 hours before harvesting the cells. l) IB analysis of WCL derived from 293T cells transfected with HA‐Cdc20, Flag‐TPD52 WT and Flag‐TPD52 K179R mutant. Where indicated, 100 µg mL^−1^ CHX was added for the indicated time period before harvesting. WT, wild type. m) The TPD52 protein abundance in (l) was quantified by ImageJ and plotted as indicated. TPD52 bands were normalized to β‐actin. n) IB analysis of WCL derived from T24 cells stably expressing shCdc20 or Scr synchronized at M phase by nocodazole block, following by releasing back into the cell cycle for the indicated times. Scr, Scramble. o) IB analysis of WCL derived from T24 cells stably expressing shCdc20 or Scr treated with Taxol (500 nM, 24 h). p) IB analysis of WCL derived from T24 Cdc20 knockdown or control cells transduced with shTPD52 lentivirus. Scr, Scramble. q‐r) Colony formation assays (q) and quantification (r) of T24/5637 Cdc20 knockdown or control cells transduced with shTPD52 lentivirus. Scr, Scramble. Error bars are mean ± s.e.m. *P < 0.05.

To explore the regulatory mechanism of TPD52 protein stability, we treated T24 cells separately with MG132 and chloroquine (CQ). Our results indicated that MG132 treatment but not CQ treatment increased the protein abundance of TPD52 in T24 cells (Figure S5d,e, Supporting Information), indicating the involvement of ubiquitin‐mediated pathways rather than autophagy‐mediated pathways in controlling TPD52 stability. Previous studies have reported that APC^FZR1^ and APC^Cdc20^ are dynamically controlled during distinct phases of the cell cycle, in which APC^FZR1^ is active during mitotic exit and G1 phase, while APC^Cdc20^ drives anaphase in early mitosis.^[^ ^30^ ^]^ Surprisingly, TPD52 interacts with both Cdc20 and FZR1 (**Figure** **5**a). We then depleted endogenous Cdc20 and FZR1 and found that Cdc20 knockdown but not FZR1 knockdown led to significant upregulation of TPD52 as well as other identified Cdc20 substrates, including Bim and p21^[^ ^31^ ^]^ (Figure 5b–d). In support of the exogenous immunoprecipitation (IP) results, the results of endogenous IP also verified the interaction between TPD52 and Cdc20 (Figure 5e). Consistent with these findings, Cdc20 depletion significantly increased the half‐life of the TPD52 protein (Figure 5f,g; Figure S5f,g, Supporting Information). We also applied apcin, a novel cell‐permeable molecule that blocks the interaction between APC/C and Cdc20,^[^ ^32^ ^]^ to analyze the regulatory effect of Cdc20 on TPD52. As expected, we found an increase in the TPD52 protein abundance upon apcin treatment (Figure S5h, Supporting Information).

We further investigated the possibility that Cdc20 ubiquitinates TPD52. Indeed, ubiquitination assays showed that Cdc20 could increase TPD52 protein ubiquitination (Figure 5h,i). To determine the ubiquitin chain linkage type(s) attached to TPD52 by Cdc20, we overexpressed wild‐type (WT) ubiquitin and single‐lysine‐ubiquitin mutants (K6‐, K11‐, K27‐, K29‐, K33‐, K48‐, and K63‐ubiquitin) to test their individual effects on TPD52 polyubiquitination and found that Cdc20 promoted K27‐, K33‐, K48‐ and K63‐linked polyubiquitination of TPD52 (Figure 5j). To determine the lysine residue(s) of TPD52 ubiquitinated by Cdc20, we applied mass spectrometry analysis of 293T cells transfected with Flag‐tagged TPD52 in combination with HA‐tagged Cdc20 and ubiquitin. Ubiquitination at lysine residues 60, 98, 152, and 179 in TPD52 was detected by mass spectrometry. We then mutated the major individual lysine residues to arginine (K60R, K98R, K152R, and K179R), and we found that mutation of K179 (but not K60, K98, or K152) decreased Cdc20‐mediated polyubiquitination of TPD52 (Figure 5k). Thus, we identified the formation of a polyubiquitin chain on TPD52 K179 mediated by Cdc20. Consistent with this finding, compared with TPD52 WT, the TPD52 K179R mutant was resistant to Cdc20‐mediated degradation (Figure 5l,m). Moreover, cells expressing the TPD52 K179R mutant exhibited reduced proliferation rates and heightened sensitivity to Tu treatment compared to both control cells and cells expressing the TPD52 wild‐type (WT) variant, primarily attributed to the increased stability conferred by the K179R mutation of TPD52 (Figure S5i,j, Supporting Information).

Consistent with these findings, depletion of Cdc20 resulted in a rather stable pattern of TPD52 expression throughout the cell cycle (Figure 5n). We also found that Cdc20 depletion attenuated the Taxol‐induced upregulation of TPD52 (Figure 5o). Functionally, depletion of TPD52 largely attenuated the increase in ER stress (Figure 5p) and promoted proliferation (Figure 5q,r; Figure S5k,l, Supporting Information) in Cdc20‐depleted cells. Overall, these results indicate that APC^Cdc20^ can bind TPD52 and mark it for degradation by promoting its polyubiquitination at K179.

### 2.6 APC^Cdc20^ Promotes TPD52 Ubiquitination in a D‐Box‐Dependent Manner

**Figure 6:**
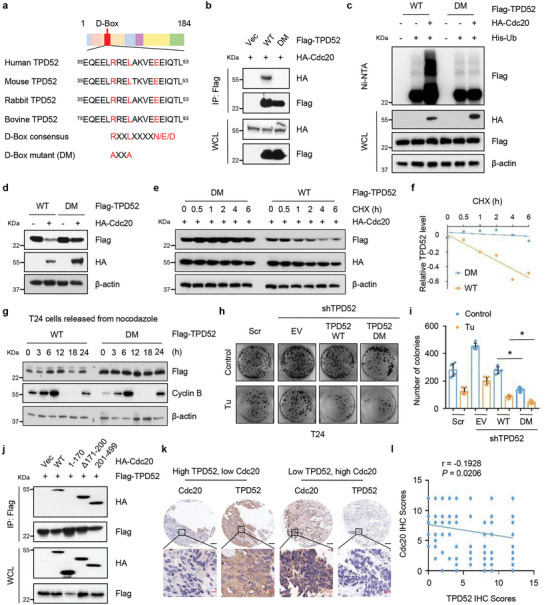
APC^Cdc20^ promotes TPD52 ubiquitination in a D‐box‐dependent manner. a) Sequence alignment of putative D‐box‐containing region between TPD52 proteins from various species as well as a schematic representation of the D‐box mutant (DM) generated and used in the following studies. b) Immunoblot (IB) analysis of whole cell lysates (WCL) and anti‐Flag immunoprecipitates (IPs) derived from 293T cells transfected with HA‐Cdc20, Flag‐TPD52 WT and Flag‐TPD52 DM mutant. 30 h post‐transfection, cells were treated with 20 µM MG132 for 6 h before harvesting. Vec, vector. WT, wild type. c) IB analysis of WCL and Ni‐NTA pull‐down products derived from 293T cells transfected with HA‐Cdc20, Flag‐TPD52 WT and Flag‐TPD52 DM mutant and His‐Ub. Where indicated, 20 µM MG132 was added for 6 hours before harvesting the cells. WT, wild type. d) IB analysis of WCL derived from 293T cells transfected with HA‐Cdc20, Flag‐TPD52 WT and Flag‐TPD52 DM mutant. WT, wild type. e) IB analysis of WCL derived from 293T cells transfected with HA‐Cdc20, Flag‐TPD52 WT and Flag‐TPD52 DM mutant. Where indicated, 100 µg mL^−1^ CHX was added for the indicated time period before harvesting. WT, wild type. f) The TPD52 protein abundance in (e) was quantified by ImageJ and plotted as indicated. g) IB analysis of WCL derived from T24 cells transfected with Flag‐TPD52 WT and Flag‐TPD52 DM mutant synchronized at M phase by nocodazole block, following by releasing back into the cell cycle for the indicated times. WT, wild type. h‐i) Colony formation assays (h) and quantification (i) of T24 TPD52 knockdown or control cells treated with Tu. T24 TPD52 knockdown cells were transfected with TPD52 WT and TPD52 DM mutant. Scr, Scramble. EV, empty vector. Error bars are mean ± s.e.m. *P < 0.05. j) IB analysis of WCL and anti‐Flag IPs derived from 293T cells transfected with Flag‐TPD52 and indicated constructs of HA‐Cdc20. Vec, vector. WT, wild type. k) Representative images of bladder cancer patient samples stained for TPD52 and Cdc20 expression by immunohistochemistry. Scale bars: 200 µm (black); 50 µm (red). l) Correlation analysis of TPD52 and Cdc20 expression in bladder cancer patient samples.

To gain further insights into how Cdc20 regulates TPD52 activity, we thoroughly investigated the specific regions of TPD52 that are recognized by Cdc20. The Co‐IP results showed that Cdc20 specifically interacted with the region between amino acids 22 and 55 in TPD52 (Figure S6a,b, Supporting Information). Cdc20 usually recognizes substrates that contain a destruction box (D‐box) motif (RXXLXXXXN/D/E^[^ ^31^, ^33^ ^]^, where R represents arginine; L represents leucine; N represents asparagine; D represents aspartate; E represents glutamate, and X represents any amino acid), and we found that TPD52 contains one evolutionarily conserved D‐box (^40^ **R**RE**L**AKVE**E** ^48^ in humans) within the 22‐55‐amino acids (aa) region (**Figure** **6**a). More importantly, mutation of the putative D‐box (to generate TPD52 DM) disrupted the interaction of TPD52 with Cdc20 (Figure 6b). Additionally, Cdc20 failed to promote TPD52 DM polyubiquitination (Figure 6c) and degradation (Figure 6d–f) compared to WT TPD52 polyubiquitination and degradation. Consistent with this finding, we were unable to detect the fluctuation pattern of TPD52 DM expression throughout the cell cycle (Figure 6g). In keeping with these findings, TPD52 DM‐expressing cells demonstrated slower proliferation and increased sensitivity to Tu compared with either control cells or TPD52 WT‐expressing cells (Figure 6h,i). Structurally, Cdc20 contains a WD40 repeat domain at its C‐terminus that mediates substrate recognition.^[^ ^34^ ^]^ As shown in Figure 6j and Figure S6c, Supporting Information, TPD52 specifically interacted with the WD40‐repeat domain of Cdc20, supporting the hypothesis that TPD52 is a putative Cdc20 substrate. To further evaluate the clinical correlation between Cdc20 and TPD52, we performed immunohistochemical (IHC) staining in bladder cancer patient samples and found a negative correlation (Rho = ‐0.1928, *P* = 0.0206) between the two proteins (Figure 6k,l). In summary, we discovered that the TPD52 D‐box motif is the binding site in TPD52 mediating its interaction with Cdc20 and subsequent degradation.

### 2.7 APC^Cdc20^ Attenuates ER Stress‐Induced Cell Death and Exerts Antitumor Effects by Repressing TPD52

**Figure 7:**
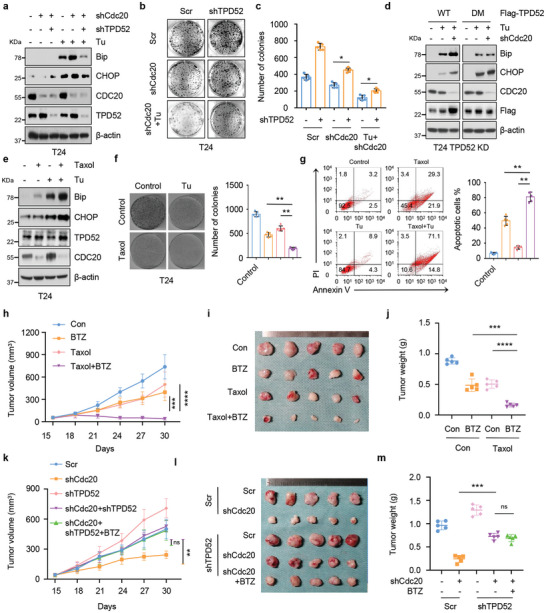
APC^Cdc20^ attenuates ER stress‐induced cell death and anti‐tumor effects through repressing TPD52. a) Immunoblot (IB) analysis of whole cell lysates (WCL) derived from T24 cells stably expressing shCdc20 or shTPD52 treated with Tu (1 µg mL^−1^, 12 h). b‐c) Colony formation assays (b) and quantification (c) of T24 cells stably expressing shCdc20 or shTPD52 treated with Tu (1 µg ml^−1^, 12 hours). Error bars are mean ± s.e.m. *P < 0.05. d) IB analysis of WCL derived from T24 TPD52 or Cdc20 knockdown cells transfected with TPD52 WT or TPD52 DM mutant under the treatment of Tu (1 µg mL^−1^, 12 h) or Taxol (500 nM, 24 h). WT, wild type. e) IB analysis of WCL derived from T24 cells treated with Tu (1 µg mL^−1^, 12 h) or Taxol (500 nM, 24 h). f) Colony formation assays and quantification of T24 cells treated with Tu (1 µM, 12 h) or Taxol (500 nM, 24 h). Error bars are mean ± s.e.m. *P < 0.05. g) Annexin‐V/PI Flow cytometry analysis and quantification of T24 cells treated with Tu (1 µg mL^−1^, 12 h) or Taxol (500 nM, 24 h). Error bars are mean ± s.e.m. *P < 0.05. h‐j. T24 cells were subcutaneously injected into nude mice to establish xenograft model and treated with Taxol (10 mg kg^−1^; twice a week) or Bortezomib (1 mg kg^−1^, twice a week). Statistical analysis of the tumor volumes which were measured every three days and plotted individually h). Subcutaneous xenograft tumors formed from different groups were dissected i). Statistical analysis of the weight of the dissected xenografts tumors j). n = 5 mice per experimental group, the results indicate the mean ± S.D. Scr, Scramble. ^***^ *P*<0.001; ^****^ *P*<0.0001. k‐m) T24 cells stably expressing shCdc20 or shTPD52 were subcutaneously injected into nude mice to establish xenograft model and treated with Bortezomib (1 mg kg^−1^, twice a week). Statistical analysis of the tumor volumes which were measured every three days and plotted individually k). Subcutaneous xenograft tumors formed from different groups were dissected l). Statistical analysis of the weight of the dissected xenografts tumors m). n = 5 mice per experimental group, the results indicate the mean ± S.D. Scr, Scramble. ^**^ *P* < 0.01; ^***^ *P* < 0.001.

Given the critical role of Cdc20 in mitotic regulation, recent studies have begun to reveal an oncogenic role for Cdc20. We next aimed to determine whether the biological significance of APC^Cdc20^‐mediated TPD52 degradation also accounts for tumorigenesis. Compared with control cells, Cdc20‐depleted cells showed higher ER stress and were more sensitive to Tu‐induced apoptosis, a phenotype that could be largely rescued by additional depletion of TPD52 (**Figure** **7**a–c). In accordance with the above findings, we found that Cdc20 knockdown cells expressing TPD52 DM displayed stable ER stress compared with cells expressing TPD52 WT under combination treatment with Tu (Figure 7d). We next tested the effect of combination treatment with Tu and Taxol, which was previously used to inhibit Cdc20. Interestingly, treatment with Taxol enhanced Tu‐induced ER stress (Figure 7e) and the antiproliferative effect of Tu in T24 cells (Figure 7f,g).

To verify the in vitro results, we constructed a subcutaneous xenograft model in nude mice. Consistent with the in vitro results, Taxol greatly decreased the volumes and weights of xenografts under ER stress inducer Tu or Bortezomib treatment conditions (Figure 7h–j; Figure S7a–f, Supporting Information). As shown in Figure 7k–m, Cdc20 depletion increased the antitumor effects, whereas additional depletion of TPD52 attenuated these effects in vivo. Consistent with this finding, the Cdc20 depletion‐mediated decreases in the volumes (Figure 7k) and weights (Figure 7m) of xenografts under Bortezomib treatment conditions were partially abolished by TPD52 depletion. The validity of these findings was further confirmed by experimental results obtained by Tu (Figure S7d–f, Supporting Information). In summary, these results support the idea that Cdc20 depletion endows bladder cancer cells with sensitivity to ER stress‐induced cellular apoptosis mainly via the reduced degradation of TPD52.

## 3 Discussion

As an important intracellular homeostatic system, the unfolded protein response (UPR), which is activated by ER stress, is essential for triggering cell apoptosis to eliminate undesired proteins and cells.^[^ ^3^, ^4^ ^]^ However, the mechanism of UPR activation in tumors remains largely elusive. Here we uncovered a novel and pivotal role of TPD52 in regulating ER stress. Specifically, TPD52 could interact with ATF6 at its luminal domain and thereby promote ATF6 cleavage. In addition, we generated *Tpd52* knockout (KO) mice and demonstrated that TPD52 promoted ER stress activation in vivo. In addition, we identified APC^Cdc20^ as a specific E3 ubiquitin ligase for TPD52 polyubiquitination. Cdc20 catalyzes the polyubiquitination of TPD52 to mark it for degradation, leading to the downregulation of ATF6 and ER stress as well as the progression of cancers. Therefore, our results demonstrate a novel Cdc20‐TPD52‐ATF6 axis involved in ER stress activation and oncogenic functions.

TPD52 has been characterized as an oncogene in multiple cancers. PrLZ, prostate‐specific isoform 1 of TPD52, has been identified to exert a tumor‐promoting effect by activating the STAT3/BCL2 pathway,^[^ ^35^ ^]^ inhibiting LKB1/AMPK,^[^ ^36^ ^]^ transactivating the androgen receptor^[^ ^37^ ^]^ and enhancing chaperone‐mediated autophagy.^[^ ^17^ ^]^ However, our results indicated that TPD52 could inhibit tumor progression via ATF6 branch of UPR in bladder cancer. In turn, accumulation of Golgi TPD52 in bladder cancer cells appears to directly result in the cleavage of ATF6. Analysis of tissue chips showed that TPD52 was downregulated in human bladder cancer tissues compared with adjacent normal tissues. In addition, loss of TPD52 promoted behaviors associated with tumor progression in vitro and in vivo. Therefore, Golgi TPD52 appears to act as a regulatory protein and inhibit tumorigenesis through the modulation of ATF6 activation. These results suggested that TPD52 played differential pathological roles in different tissues and multifarious binding proteins might be the molecular mechanism accounts for orchestrating cancer phenotypes.

The anaphase‐promoting complex/cyclosome (APC/C, also named APC) regulates mitosis by targeting specific substrates for degradation during the cell cycle.^[^ ^30^ ^]^ The APC core subunits form a platform for the recruitment of the substrate‐recruiting proteins Cdc20 and Fizzy‐related protein 1 (FZR1, also named Cdh1). Previous studies have revealed an oncogenic role for Cdc20 through mediating a majority of tumor suppressor destruction. In addition, studies have shown that targeting Cdc20 may be an effective therapeutic approach for cancer.^[^ ^32^, ^38^ ^]^ Structural studies have shown that Cdc20 can selectively bind to TPD52 by recognizing the D‐box motif (^40^RRELAKVEE^48^ in humans). Because PrLZ contains the same C‐terminal domain (including the D‐box motif) as TPD52, PrLZ can also bind to Cdc20 and FZR1 (data not shown). A previous study identified SPOP as the specific cytoplasmic E3 ubiquitin ligase of PrLZ and Cdc20.^[^ ^39^, ^40^ ^]^ Interestingly, SPOP was also found to be a bona fide FZR1 substrate.^[^ ^41^ ^]^ This finding led us to identify which APC mediates the regulation of PrLZ ubiquitination and investigate the regulatory mechanism linking these proteins in prostate cancer.

The UPR has a critical role in cancer cell death and homeostasis regulation, and pharmacological agents that activate the UPR can induce cell death by increasing the levels of apoptosis‐inducing proteins, such as CHOP and ATF6.^[^ ^42^ ^]^ During the UPR, ATF6 is released from the ER and subsequently transported to the Golgi, where it is cleaved by S1P and S2P. Interestingly, we found that TPD52 bound to S2P, thus promoting the ATF6‐S2P interaction and ATF6 cleavage. In contrast, PrLZ could not interact with S2P. It has been demonstrated that TPD52 contains the same C‐terminal domain (amino acids 7–184) as the PrLZ protein, and we further sought to determine the specific molecular mechanism underlying their functional differences. We found that the TPD52‐N‐terminus, i.e., amino acids 1–6, specifically mediated TPD52 binding to S2P. Deletion of amino acids 1–6 of TPD52 disrupted the interaction of TPD52 with S2P. TPD52 has an N‐terminal domain different from that of the PrLZ protein, which explains why these two isoforms display different functions in specific organs. In addition, we clarified that TPD52 activates the UPR by promoting ATF6 cleavage and increasing the antitumor activity of tunicamycin. Due to the lack of small molecule‐binding sites in ATF6, developing compounds to target ATF6 is extremely challenging. Thus, our study verifies the possibility of applying a specific Cdc20 inhibitor to increase the abundance of the TPD52 protein. We found that treatment with Cdc20 inhibitors, such as apcin and Taxol, significantly reduced TPD52 polyubiquitination and degradation and proved to have an excellent synergistic effect with tunicamycin in cancer treatment. Taken together, our findings demonstrated a molecular rationale for combining a Cdc20 inhibitor with a UPR activator and offered a novel strategy for cancer therapy.

## 4 Experimental Section

### Cell Culture and Transfection and Viral Infection

Human bladder cancer cell lines T24, 253J, J82, SW780, and 5637, melanoma cell line A375, and human embryonic kidney cell line HEK293T were obtained from ATCC and were cultured in a 5% CO_2_ incubator at 37 °C. SW780, 5637, and A375 were maintained in RPMI 1640 medium supplemented with 10% fetal bovine serum (FBS), 100 µg mL^−1^ penicillin, and 100 µg mL^−1^ streptomycin. T24, J82, and 253J were cultured in DMEM supplemented with 10% FBS, 100 µg mL^−1^ penicillin and streptomycin. For transfection, plasmid and polyethyleneimine (PEI) were diluted in Opti‐MEM medium in a ratio of 1:3 (ug DNA: ug PEI). After incubated at room temperature for 15 min, the mixture was carefully transferred to the prepared cells. 48 hours after transfection, samples are collected for further experiment. For viral infection, psPAX2 packaging plasmid and pMD2G envelope plasmid were used for lentiviral packaging in HEK‐293T cells according to the manufacturer's protocol. 48 h after transfection, supernatant was harvested and used to infect cells. Puromycin was used for cell selecting, and unstably infected cells were removed.

### Reagents, Antibodies, and Plasmids

Tunicamycin (Tu, HY‐A0098), nocodazole (HY‐13520), apcin (HY‐110287), thymidine (HY‐N1150), and paclitaxel (Taxol, HY‐B0015) were purchased from MedChemExpress (New Jersey, NJ, USA). Anti‐Flag agarose beads (A‐2220) and anti‐HA agarose beads (A‐2095) were purchased from Sigma‐Aldrich (St. Louis, MO, USA). Rabbit primary antibodies against ATF6 (65 880), eIF2α (5324), phospho‐eIF2α (Ser 51) (3398), PERK (3192), IRE1α (3294), Bip (3177), cyclin D1 (2978), Flag‐tag (14 793), HA‐tag (3724), GST‐tag (2625) and β‐actin (4970) were all purchased from Cell Signaling Technology, Inc (Boston, MA, USA). Antibodies against phospho‐IRE1α (S724) (ab48187), TPD52 (ab182578), Bim (ab32158) and p21 (ab109520) were purchased from Abcam (Cambridge, MA, USA). Antibodies against cyclin B (sc‐245), cyclin E (SC‐247), FZR1 (sc56312), Cdc20 (sc‐8358), Cdc20 (sc‐13162) were obtained from Santa Cruz (California, CA, USA). Rabbit polyclonal PrLZ antibody was offered from Professor Ruoxiang Wang (Department of Medicine, Cedars‐Sinai Medical Center, Los Angeles, CA, USA). Anti‐rabbit IgG peroxidase antibody produced in goat (A9169;) or anti‐mouse IgG peroxidase antibody produced in goat (A4416) were purchased from Sigma‐Aldrich (St. Louis, MO, USA). The enhanced chemiluminescence (ECL) detection system (1 705 062) was from Bio‐Rad Laboratories, Inc. (Hercules, CA, USA). The pCMV‐3xFlag‐ATF6 (11 975), pFLAG‐TPD52 (172 452), and pCMV‐8xHis‐Ub (107 392) was purchased from Addgene. pcDNA3‐HA‐TPD52, pcDNA3‐HA‐PrLZ, pcDNA3‐HA‐FZR1, pcDNA3‐HA‐Cdc20, pGEX‐4T‐1‐GST‐TPD52, and His‐Ubiquitin K‐only mutants (K6/K11/K27/K29/K33/K48/K63) plasmids were constructed according to standard protocols in our laboratory. Different TPD52, ATF6, and Cdc20 mutants were generated according to the manufacturer's instructions as described before(17, 39). pLKO‐shATF6, pLKO‐shTPD52, pLKO‐shCdc20, and pLKO‐shFZR1 were all purchased from Sigma‐Aldrich (St. Louis, MO, USA).

### Cell Viability Assay

Cells were planted into 96‐well plates (1.0×10^4^ cells per well), and added Thiazolyl Blue (MTT, TargetMol T19029) after treatment. After incubating with MTT for 4 h, cells were lysed with dimethyl sulfoxide (DMSO). The cell viability was determined with a 96‐well microplate reader (Bio‐Rad, Hercules, CA) at wavelength of 490 nm. The experiments were performed in triplicate.

### Colony Formation Assay

Cells were seeded into a 6‐well plate (1.0×10^3^ cells per well) and incubated with different treatments. One week later, the cells were washed with phosphate‐buffered saline (PBS) and fixed with 4% paraformaldehyde for 15 min. The cells were then stained with crystal violet solution for 15 min. The staining solution was slowly washed away with running water, and then photographed with inverted microscope. All the colony formation assays were repeated at least three times.

### Transwell Migration and Invasion Assay

Cells were resuspended with serum‐free medium. For migration assay, cells were added to the upper chamber and DMEM medium containing 10% FBS was added to the lower chamber. For invasion assay, a mixture of serum‐free DMEM/Matrigel (Sigma‐Aldrich; Merck KGaA) was plated onto the upper chamber. After incubating for 24 or 48 h, transwell plates were fixed with 4% paraformaldehyde and stained with crystal violet solution (0.1%, dissolved in ethanol). An inverted microscope was used to take photos in 5 randomly selected fields (magnification, x100). All the migration and invasion assays were repeated at least three times.

### Tandem Fluorescence Microscopy and Immunofluorescence Staining

Bladder cancer cells T24 were seeded onto round cover slips and fixed with paraformaldehyde (4%) at room temperature. After permeabilized with Triton X‐ 100(0.5%) for 15 min and blocked with fetal bovine serum (5%) for 1 h, the cells subsequently incubated with an anti‐ATF6 primary antibody (1:100) overnight at 4 °C. Then the cells were washed with PBS and incubated with fluorescent labeled secondary antibodies at room temperature for 1 h. The localization of immunofluorescence signals was observed under Tandem fluorescence microscopy after sealed with glycerol on slides.

### Co‐Immunoprecipitation

Briefly, the cells were transfected with particular plasmids and incubated for 48 h. Then cells were lysed with IP buffer [50 mM Tris HCl, 150 mM NaCl, 1 mM ethylenediaminetetraacetic acid (EDTA), and 1% Triton X‐100] containing protease inhibitors and phosphatase inhibitors. The proteins were incubated with antibody‐conjugated M2 agarose beads with gentle rocking at 4 °C for 4 h. Later, the cell lysates were washed extensively with IP buffer, and the proteins were extracted from the Dynabeads by boiling at 95 °C for 5 min. Finally, the proteins were separated on SDS‐PAGE for western blotting.

### Total RNA Extraction and Real‐Time RT‐PCR Analysis

Total cell RNA was extracted using TRIzol reagent (Invitrogen; Thermo Fisher Scientific) according to the manufacturer's instructions. Primer Script RT reagent kit (Takara Bio) was used for cDNA synthesis. cDNA was diluted in SYBR Green Master Mix (Takara Bio) and then real‐time RT‐PCR was conducted following cycling conditions: 95 °C for 15 s, 55 °C for 30 s, and 72 °C for 30 s. Relative mRNA levels were evaluated using the 2^−ΔΔ^ CT method. The primer sequences are listed in Table S1.

### GST Pull‐Down Assay

Purified His‐Flag tagged ATF6 protein was incubated with GST‐TPD52 protein immobilized by glutathione agarose beads in the binding buffer (50 mM Tris‐HCl, pH [7.5], 1% Triton X‐100, 150 mM NaCl, 0.5 mM EDTA). After rotating at 4 °C for ≈4 h, the glutathione agarose beads were washed with binding buffer for three times. Then the beads were boiled at 95 °C for 5 min in SDS sample buffer and analyzed by western blotting.

### In Vivo Ubiquitination Assays

HEK293T cells were transfected with His‐ubiquitin and indicated plasmids using PEI‐based transfection method. After 48 h, cells were lysed using Buffer A (6 M guanidine HCl, 0.1 M Na_2_HPO_4_/NaH2PO_4_, 10 mM imidazole, pH 8.0) on ice, and centrifuged at 10 000 g for 30 s. The supernatant lysate was incubated with Ni‐NTA beads at room temperature. 4 h later, the beads were centrifuged and washed with sequential Buffer A, Buffer B (8 M urea, 0.1 M Na_2_HPO_4_/NaH2PO_4_ pH 8.0, 0.01 M Tris‐HCl pH 8.0, 10 mM β‐Mercaptoethanol) and Buffer C (8 M URA, 0.1 M Na_2_HPO_4_/NaH2PO_4_ pH 6.3, 0.01 M Tris‐HCl, pH 6.3, 10 mM β‐Mercaptoethanol, 0.2% Triton X‐100) separately. Then Ni‐NTA beads were boiled at 95 °C for 5 min in SDS sample buffer and analyzed by western blotting.

### Mass Spectrometry Analysis

To identify the potential interacting proteins of TPD52, T24 human bladder cancer cells were lysed with IP buffer and the supernatant lysates were immunoprecipitated with TPD52 antibody. Then the immunoprecipitates were eluted with SDS sample buffer and separated in a 12% acrylamide gradient gel. Gel‐resolved proteins were enzymatically digested with trypsin to generate peptides that are suitable for subsequent analysis. Then C18 Stage Tips (Thermo Fisher Scientific) was used to de‐salt peptides to remove impurity and undigested proteins. Finally, the preprocessed sample was redissolved in 0.1% formic acid and analysed using Liquid 531 Chromatography with tandem mass spectrometry (LTQ‐Orbitrap, Thermo 532 Fisher Scientific). LC/MS data were compared against the Uniprot protein database using Mascot 2.5.1 (Matrix Science) and then analyzed using the Scaffold 4.4.8 software (Proteome Software). Peptides were accepted if they passed a 1% FDR threshold. GO enrichment analysis was performed using DAVID database (https://david.ncifcrf.gov/). To detect ubiquitination sites of TPD52, HEK293T cells were transfected with HA‐TPD52 and Flag‐CDC20 for the in vivo ubiquitination reaction. The ubiquitinated HA‐TPD52 was immunoprecipitated with HA‐ agarose beads. After digested with trypsin, the peptides following in vivo ubiquitination assay were scanned with a mass spectrometer to determine ubiquitin‐modified sites.

### Cell Synchronization

Cancer cells were synchronized using double thymidine/ Nocodazole arrest and release or using serum starvation and refeed. Then cells were collected at different time points for further experiments.

### Construction of *Tpd52*‐Knockout Mice and Tissue Extraction

*Tpd52*‐knockout mice were generated as described before.^[^ ^17^ ^]^ In brief, a pair of LoxP sites was initially inserted into intron regions flanking TPD52 exon2, and germline chimeric mice were generated. Subsequently, these mice were crossed with CMV‐Cre transgenic mice (F1 generation), and its offspring that possessed both the heterozygous allele and the CMV‐Cre transgene were bred with heterozygous mice (F2 generation). This breeding scheme resulted in obtaining F3 generation offspring that are homozygous for the allele and heterozygous for the CMV‐Cre transgene, representing the mutants. Homozygous *Tpd52* KO mice were confirmed by PCR genotyping. All operations were performed according to the NIH Guidelines on the Use of Laboratory Animals. C57BL/6 mouse experiments were performed using protocols approved by the Institutional Animal Care and Use Committee of Xi'an Jiaotong University. Different tissue of C57BL/6 mice were isolated and used for protein extraction. For chemical carcinogen induced skin tumors, the shaved naked abdomen of WT or *TPD52* ^−/‐^ mice were painted with 150 µg DMBA (D3254; Sigma‐Aldrich, MO, USA) resolved in 200 µL acetone. Two weeks later, the mice were treated with 20 µg TPA (HY‐18739, MedChemExpress, NJ, USA) in 200 µl acetone for 10 weeks (twice per week). The number and volumes of neoplasm were monitor for several days, and then the mice were euthanized and dissected for HE and IHC staining.

### Mouse Xenograft Assays

Balb/c nude mice were purchased from Laboratory Animal Center of Xi'an Jiaotong University (Xi'an, China), and the animal welfare was guaranteed during the experiment. For the xenograft assay, Balb/c nude mice were injected subcutaneously with gene stable knockdown T24 cells and corresponding control cells which suspended in 100 µL sterile PBS (5×10^6^cells). Two weeks after tumor cell implantation, the mice were received Tu (0.5 mg kg^−1^, once a week) or Taxol (30 mg kg^−1^, once a week) treatment, and tumor volumes were measured every three days using a vernier caliper (Volume (mm^3^) = length×width2 ×1/2). After a period of one month, the mice were euthanized and the tumor were harvested for the following experiments.

### Quantification and Statistical Analysis

All statistical analyses were performed using SPSS 15.0 (SPSS Inc, Chicago, IL) and quantitative data are presented as mean ± SE. For each experiment, three technical replicates were conducted. The differences between various groups were analyzed by one‐way ANOVA and the differences between two groups were analyzed by Student's t‐tests. *P*<0.05 was the criterion used to represent a significant difference.

## Conflict of Interest

The authors declare no conflict of interest.

## Supporting information



Supporting Information

## Data Availability

The data that support the findings of this study are available in the supplementary material of this article.
