# Supplementary material for: The Tumor Suppressor TPD52‐Governed Endoplasmic Reticulum Stress is Modulated by APCCdc20
Source: Adv Sci (Weinh). 2024 Oct 14;11(45):2405441. doi: 10.1002/advs.202405441 (PMC11615746; doi:10.1002/advs.202405441)
Supplement: Supplementary file 1 — Supporting Information [file ADVS-11-2405441-s001.docx]

Supplementary Figures for

**The tumor suppressor TPD52-governed endoplasmic reticulum stress is modulated by APC^Cdc20^**

Weichao Dan^1,2,3,4^, Yizeng Fan^1,2,3,4^, Yuzhao Wang^1,2,3,4^, Tao Hou^1,2,3^, Yi Wei^1,2,3^, Bo Liu^1,2,3^, Mengxing Li^1,2,3^, Jiaqi Chen^1,2,3^, Qixiang Fang^1,2,3^, Taotao Que^1,2,3^, Yuzeshi Lei^1,2,3^, Chendong Guo^1,2,3^, Chi Wang^1,2,3^, Yang Gao^1,2,3^, Jin Zeng^1,2,3^, and Lei Li^1,2,3,*^

^1^ Department of Urology, The First Affiliated Hospital of Xi'an Jiaotong University, Xi'an 710061, P. R. China

^2^ Key Laboratory for Tumor Precision Medicine of Shaanxi Province, The First Affiliated Hospital of Xi'an Jiaotong University, Xi’an 710061, P.R. China

^3^ Key Laboratory of Environment and Genes Related to Diseases, Ministry of Education, Xi'an 710061, P.R. China

^4^ These authors contributed equally to this work

^*^ Correspondence and requests for materials should be addressed to L.L. (E-mail: [lilydr@163.com)](mailto:lilydr@163.com))

**This file includes:**

Supplementary Figures 1-6

Supplementary Table 1

**
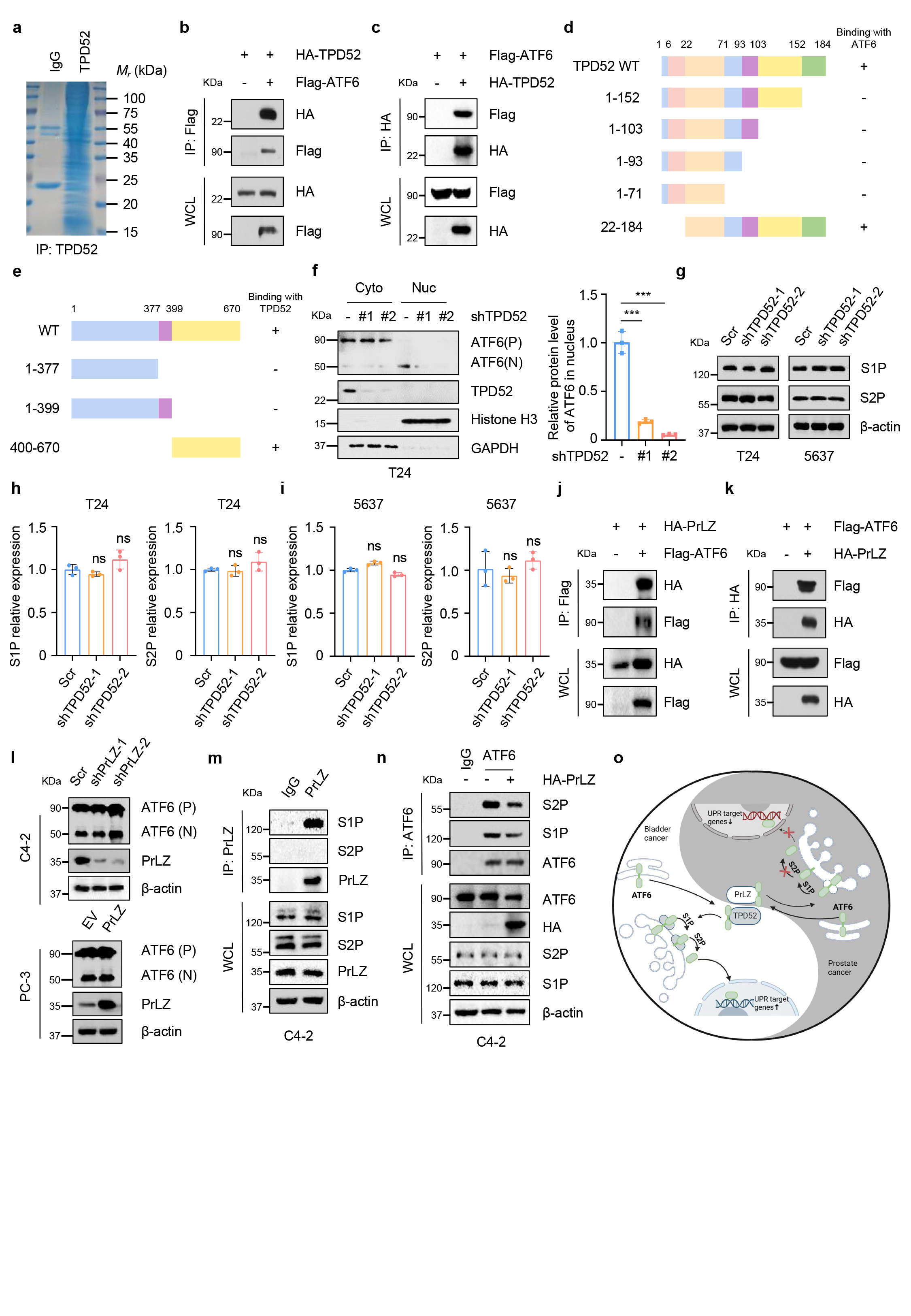
**

**Figure S1. TPD52 promotes ATF6 activation during the UPR. a.** Proteins co-immunoprecipitated by IgG and TPD52 antibody from T24 cells were subjected to SDS-PAGE gel and stained by Coomassie Blue. **b.**  Immunoblot (IB) analysis of whole cell lysates (WCL) and anti-Flag immunoprecipitates (IPs) derived from 293T cells transfected with Flag-ATF6 and HA-TPD52. **c.** IB analysis of WCL and anti-HA IPs derived from 293T cells transfected with Flag-ATF6 and HA-TPD52. **d-e.** Schematic representation of truncated constructs of TPD52 **(d)** and ATF6 **(e)** for mapping the interaction domain**. f.** IB analysis of cytoplasmic and nucleus fraction derived from T24 cells stably expressing shTPD52 or shScr. Cells were treated with Tu (1 μg ml^-1^) for 12 h before harvesting. Scr, Scramble. **g.** IB analysis of WCL derived from T24 and 5637 cells stably expressing shTPD52 or shScr. Scr, Scramble. **h-i.** mRNA level of S1P or S2P evaluated by qPCR in T24 cells **(h)** and 5637 **(i)** cells stably expressing shTPD52 or shScr. Scr, Scramble. **j.** IB analysis of WCL and anti-Flag IPs derived from 293T cells transfected with Flag-ATF6 and HA-PrLZ. **k.** IB analysis of WCL and anti-HA IPs derived from 293T cells transfected with Flag-ATF6 and HA-PrLZ. **l.** IB analysis of WCL derived from C4-2 cells stably expressing shPrLZ or shScr, and PC-3 cells stably overexpressing PrLZ or EV. Scr, Scramble. EV, empty vector. ATF6 (P) and (N), respectively, refer to ATF6 precursor and cleavaged form. **m.** IB analysis of WCL and anti-PrLZ IPs derived from C4-2 cells. **n.** IB analysis of WCL and anti-ATF6 IPs derived from C4-2 cells stably overexpressing PrLZ or EV. Where indicated, 1 μg ml^-1^ Tu was added before harvesting the cells. EV, empty vector. **o.** Schematic models of the proposed mechanism. TPD52 enhances ATF6 cleavage by facilitating interaction of S2P-ATF6 during ER stress, while PrLZ fails to interact with S2P and PrLZ overexpression results in loss of the ATF6-stimulative activity.


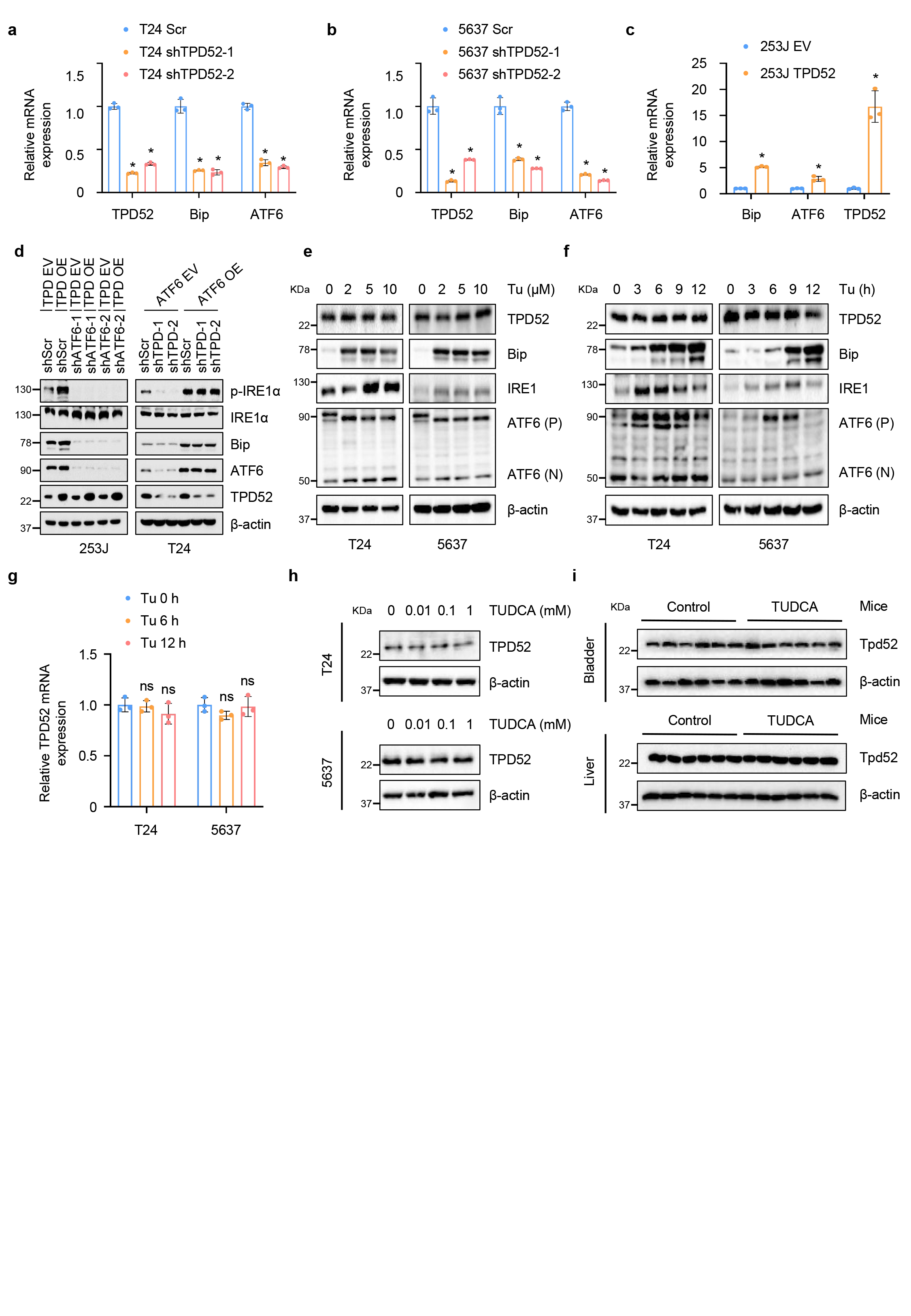


**Figure S2. TPD52 remain constant during ER stress. a-b.** mRNA level of TPD52, Bip and ATF6 evaluated by qPCR in T24 **(a)** and 5637 **(b)** cells stably expressing shTPD52 or shScr. Scr, Scramble. **c.** mRNA level of TPD52, Bip and ATF6 evaluated by qPCR in 253J cells stably overexpressing TPD52 or EV. EV, empty vector. **d.** Immunoblot (IB) analysis of whole cell lysates (WCL) from T24 or 253J cells stably expressing indicated plasmids. **e.** Immunoblot (IB) analysis of whole cell lysates (WCL) derived from T24 and 5637 cells treated with tunicamycin (Tu) at the indicated concentrations for 12 h. **f.** IB analysis of WCL derived from T24 and 5637 cells treated with 1 μg ml^-1^ Tu for indicated time. **g.** mRNA level of TPD52 in T24 and 5637 cells treated with 1 μg ml^-1^ Tu for 0, 6 and 12 h. **h.** IB analysis of TPD52 expression in T24 and 5637 cells treated with TUDCA at the indicated concentrations for 24 h. **i.** IB analysis of TPD52 expression in bladder and liver tissues of C57BL/6 mice treated with TUDCA (100 mg/kg, intraperitoneal injection once a day for 2 weeks).


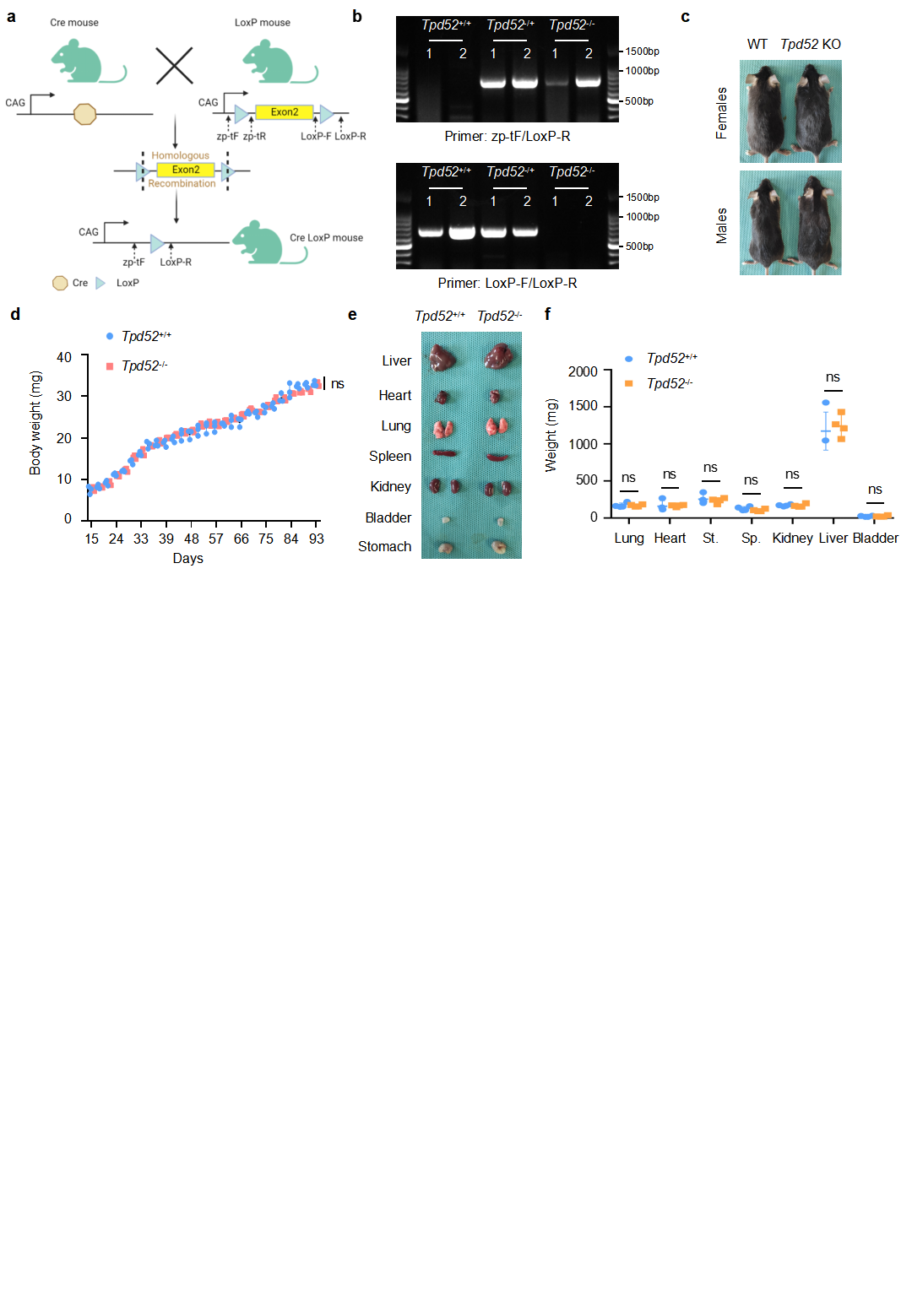


**Figure S3. Construction strategy and growth parameters of *Tpd52* knockout (KO) mice. a.** Schematic diagram of the Cre/LoxP homologous recombination strategy for targeted disruption of TPD52. **b.** PCR detection of knockout alleles. In the KO allele, a Loxp-F binding site is removed, allowing us to detect WT, heterozygote, and homozygote animals. **c.** Appearance of 12-weeks-old mice. **d.** Growth curves of WT (n=5) and *Tpd52* KO (n=5) mice. ns, no significance. **e.** Appearance of visceral organ of WT and *Tpd52* KO mice. **f.** Weights of visceral organs in of WT (n=4) and *Tpd52* KO mice (n=4). ns, no significance.


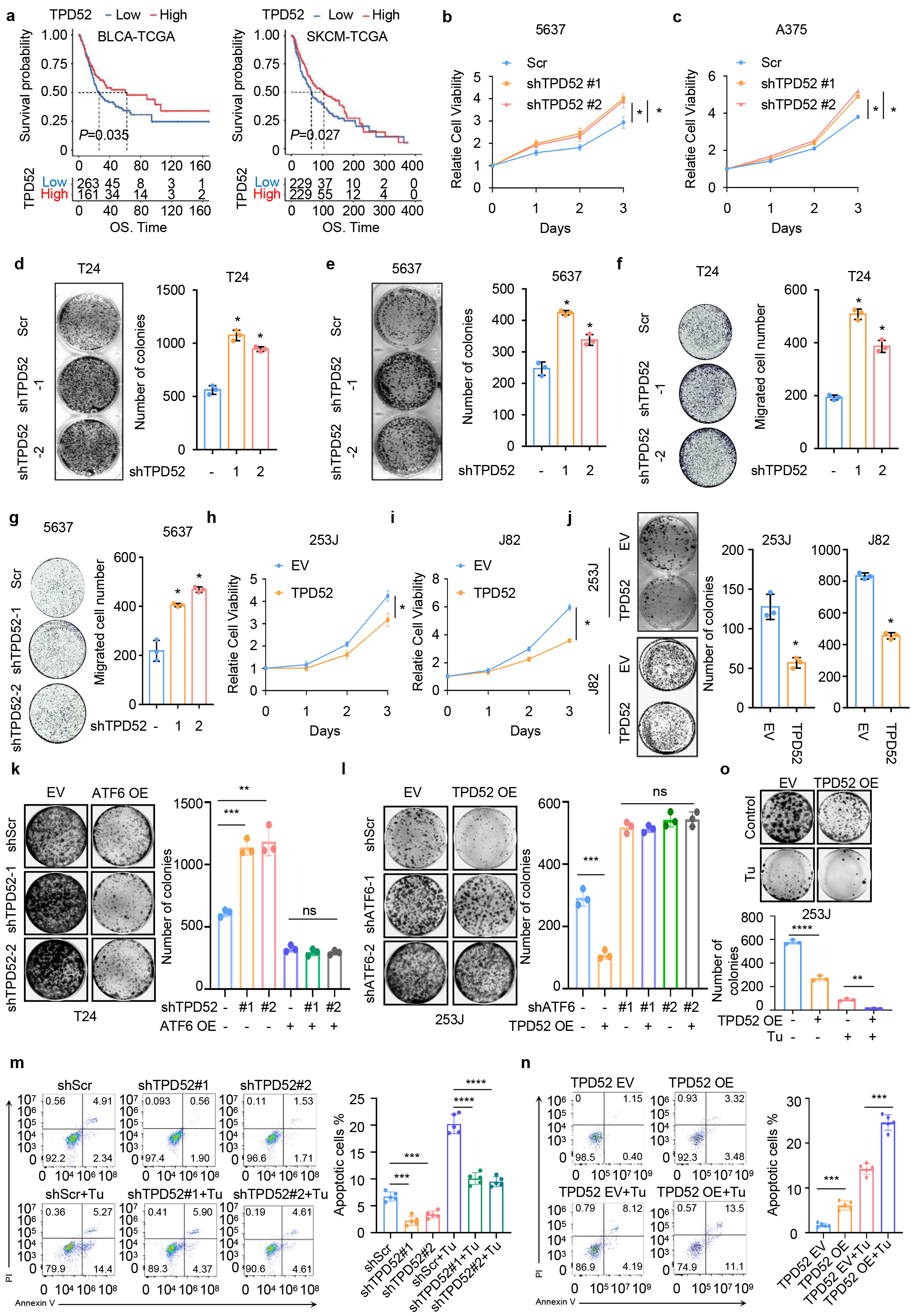


**Figure S4. TPD52-mediated ER stress inhibits tumorigenesis. a.** Prognostic significance of TPD52 in bladder cancer (BLCA) and skin cutaneous melanoma (SKCM) analyzed using TCGA database. **b-c.** The growth curve of 5637 **(b)** and A375 **(c)** TPD52 knockdown or control cells. Scr, Scramble. **P* < 0.05. **d-e.** Colony formation assays and quantification of T24 **(d)** and 5637 **(e)** cells with knockdown of TPD52. Scr, Scramble. Error bars represent SEs. **P* < 0.05. **f-g.** Transwell migration assays and quantification of migrated T24 **(f)** and 5637 **(g)** cells with knockdown of TPD52. Scr, Scramble. Error bars represent SEs. **P* < 0.05. **h-i.** The growth curve of 253J **(h)** and J82 **(i)** cells stably overexpressing TPD52 or EV. EV, empty vector. **P* < 0.05. **j** Colony formation assays and quantification of 253J and J82 cells stably overexpressing TPD52 or EV. EV, empty vector. **P* < 0.05. **k**. T24 cells was stably expressed indicated plasmids and treated with sustained low dose Tu (10 ng/ml), and colony formation assays were conducted and quantified. ***P* < 0.01; ****P* < 0.001. **l**. 253J cells was stably expressed indicated plasmids and treated with sustained low dose Tu (10 ng/ml), and colony formation assays were conducted and quantified. ****P* < 0.001. **m**. Annexin-V/PI Flow cytometry analysis and quantification of control or TPD52 knockdown T24 cells treated with Tu (1 μg ml^-1^, 12 hours). Error bars are mean ± s.e.m. ****P* < 0.001; *****P* < 0.0001. **n**. Annexin-V/PI Flow cytometry analysis and quantification of control or TPD52 overexpressing 253J cells treated with Tu (1 μg ml^-1^, 12 hours). Error bars are mean ± s.e.m. ****P* < 0.001. **o**. Colony formation assays and quantification of 253J TPD52 overexpressing or control cells treated with Tu. Error bars are mean ± s.e.m. ***P* < 0.01; *****P* < 0.0001


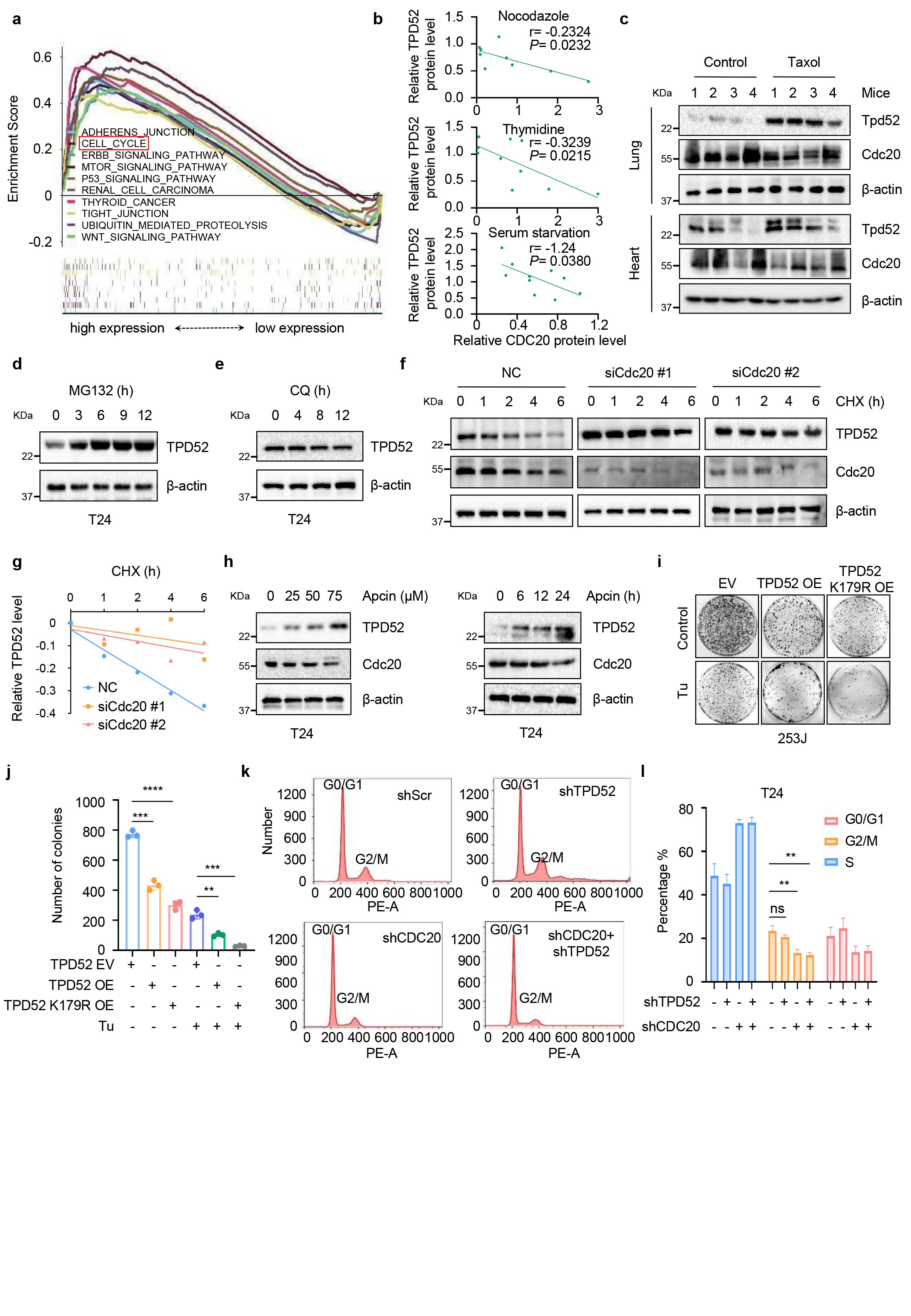


**Figure S5. The master cell cycle regulator Cdc20 regulates TPD52 degradation. a.** GSEA enrichment analysis for key biological processes associated with TPD52 in bladder cancer. **b.** Quantification of CDC20 and TPD52 blot intensity using the ImageJ software, according to Figure 4a-c. CDC20 and TPD52 bands were normalized to β-actin. **c.** Immunoblotting (IB) analysis of TPD52 and Cdc20 expression in lung and heart tissues of C57BL/6 mice treated with taxol (10 mg/kg) for 7 days. **d.** IB analysis of TPD52 expression in T24 cells treated with MG132 (10 μM) for indicated time (3, 6, 9 or 12 hours). **e.** IB analysis of TPD52 expression in T24 cells treated with CQ (chloroquine, 20 μM) for indicated time (4, 8 or 12 hours). **f.** Cdc20 knockdown cells (siCdc20) as well as parental T24 cells (NC) were treated with 100 μg/ml cycloheximide (CHX) for the indicated time period before harvesting. Equal amounts of WCL were immunoblotted with the indicated antibodies. NC, negative control. **g.** The TPD52 protein abundance in **(f)** was quantified by ImageJ and plotted as indicated. TPD52 bands were normalized to β-actin. **h.** IB analysis of TPD52 and Cdc20 expression in T24 cells treated with Apcin at the indicated concentrations for 24 hours or treated with Apcin (20 μM) for indicated time (6, 12 and 24 hours). **i-j**. Colony formation assays(**i**) and quantification(**j**) of 253J cells overexpressing indicated plasmids and treated with Tu. Error bars are mean ± s.e.m. **k-l**. Cell cycle analysis of T24 cells stably expressing shCdc20 or shTPD52.


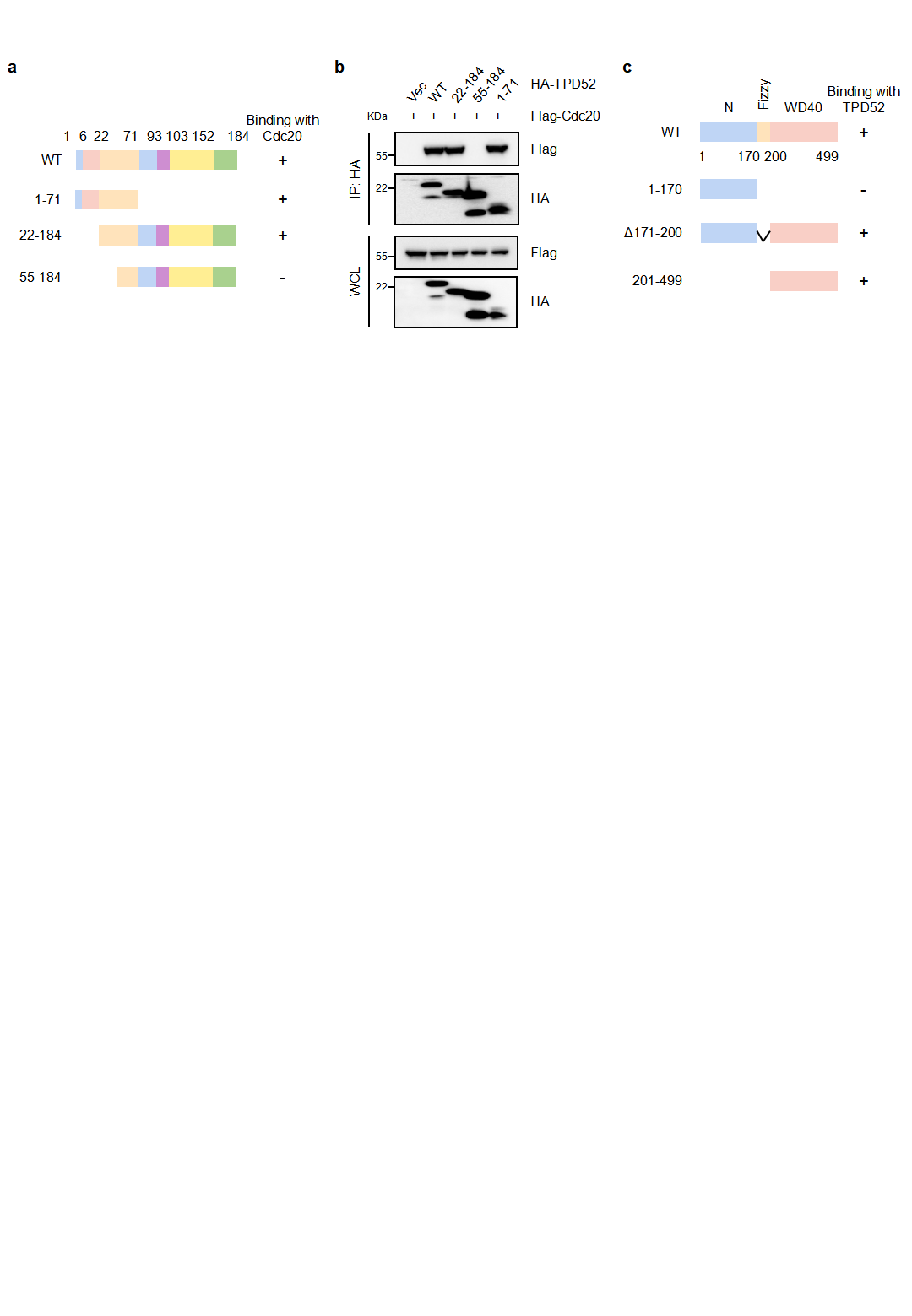


**Figure S6. Interaction domain between TPD52 and Cdc20. a.** Schematic representation of truncated constructs of TPD52 for mapping the interaction domain with Cdc20**. b.** Immunoblot (IB) analysis of whole cell lysates (WCL) and anti-HA immunoprecipitates (IPs) derived from 293T cells transfected with Flag-Cdc20 and indicated constructs of TPD52. **c.** Schematic representation of truncated constructs of Cdc20 for mapping the interaction domain with TPD52**.**

**
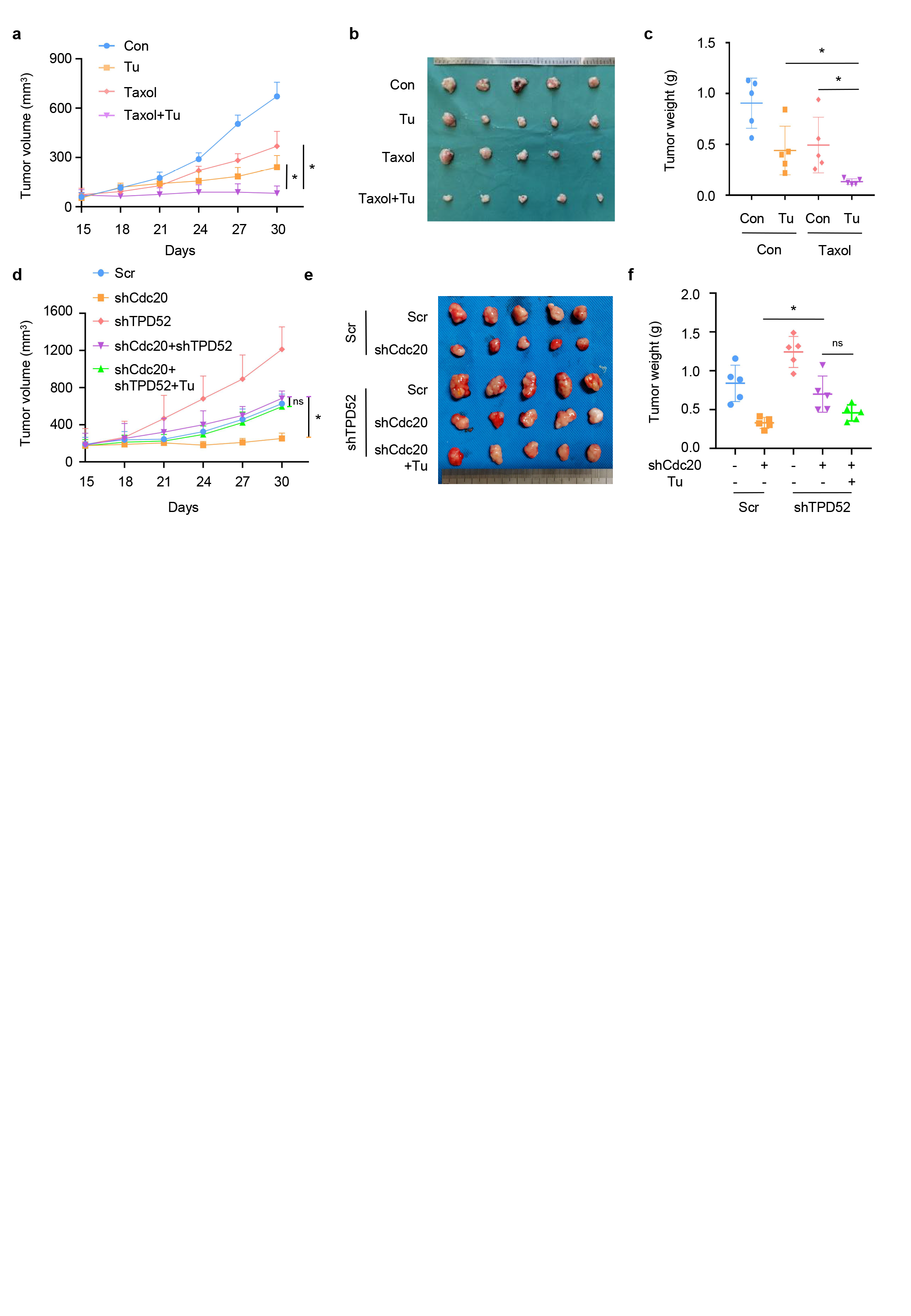
**

**Figure S7. APC^Cdc20^ inhibition enhances the cancer cell sensitivity to ER stress-induced cell death via stabilization of TPD52. a-c.** T24 cells were subcutaneously injected into nude mice to establish xenograft model and treated with taxol (10 mg/kg; twice a week) or Tu (10 mg/kg, once daily). Statistical analysis of the tumor volumes which were measured every three days and plotted individually **(a)**. Subcutaneous xenograft tumors formed from different groups were dissected **(b)**. Statistical analysis of the weight of the dissected xenografts tumors **(c)**. n = 5 mice per experimental group, the results indicate the mean ± S.D. Scr, Scramble. ^*^*P*＜0.05. **d-f.** T24 cells stably expressing shCdc20 or shTPD52 were subcutaneously injected into nude mice to establish xenograft model and treated with Tu (10 mg/kg, once daily). Statistical analysis of the tumor volumes which were measured every three days and plotted individually **(d)**. Subcutaneous xenograft tumors formed from different groups were dissected **(e)**. Statistical analysis of the weight of the dissected xenografts tumors **(f)**. n = 5 mice per experimental group, the results indicate the mean ± S.D. Scr, Scramble. ^*^*P*＜0.05.

Supplementary Table S1. List of primers.

|  | Forward primers | Reverse primers |
| --- | --- | --- |
| qRT-PCR |  |  |
| Human TPD52 | AGCATCTAGCAGAGATCAAGCG | AGCCAACAGACGAAAAAGCAG |
| Human ATF6 | TCCTCGGTCAGTGGACTCTTA | CTTGGGCTGAATTGAAGGTTTTG |
| Human GAPDH | GAGTCAACGGATTTGGTCGT | TTGATTTTGGAGGGATCTCG |
| Human S1P | ACCTCGAAACAATCCATCCAGT | ACTTGAGGGAACGAAAGACTTTT |
| Human S2P | TGGACTGTCGTCTACCTGACC | AGCAGTTTGCCATCTTATGTGG |
| Human Bip | CATCACGCCGTCCTATGTCG | CGTCAAAGACCGTGTTCTCG |
| shRNA | Forward oligo | Reverse oligo |
| hshTPD52-1 | CCGGGAAGAGCTAAGAAGAGAACTTCTCGAGAAGTTCTCTTCTTAGCTCTTCTTTTTG | AATTCAAAAAGAAGAGCTAAGAAGAGAACTTCTCGAGAAGTTCTCTTCTTAGCTCTTC |
| hshTPD52-2 | CCGGGCTTACAAGAAGACATCTGAACTCGAGTTCAGATGTCTTCTTGTAAGCTTTTTG | AATTCAAAAAGCTTACAAGAAGACATCTGAACTCGAGTTCAGATGTCTTCTTGTAAGC |
| hshTPD52-3 | CCGGGCTAAGAAGAGAACTTGCAAACTCGAGTTTGCAAGTTCTCTTCTTAGCTTTTTG | AATTCAAAAAGCTAAGAAGAGAACTTGCAAACTCGAGTTTGCAAGTTCTCTTCTTAGC |
| hshCdc20-1 | CCGGTGGTGGTAATGATAACTTGGTCTCGAGACCAAGTTATCATTACCACCATTTTTG | AATTCAAAAATGGTGGTAATGATAACTTGGTCTCGAGACCAAGTTATCATTACCACCA |
| hshCdc20-2 | CCGGCCCATTACAAGGAGCTCATCTCTCGAGAGATGAGCTCCTTGTAATGGGTTTTTG | AATTCAAAAACCCATTACAAGGAGCTCATCTCTCGAGAGATGAGCTCCTTGTAATGGG |
